# Age-Dependent Prebiotic Effects of Soluble Corn Fiber in M-SHIME^®^ Gut Microbial Ecosystems

**DOI:** 10.1007/s11130-023-01043-z

**Published:** 2023-01-25

**Authors:** Marta Calatayud Arroyo, Ieva Laurie, Chloë Rotsaert, Massimo Marzorati, Davide Risso, Kavita Karnik

**Affiliations:** 1 ProDigest, Technologiepark 82, 9052 Zwijnaarde, Belgium; 2 Center for Microbial Ecology and Technology (CMET), Faculty of Bioscience Engineering, Ghent University, Coupure Links 653, 9000 Ghent, Belgium; 3 Tate & Lyle PLC, 5 Marble Arch, W1H 7EJ London, UK

**Keywords:** Elderly, Infant, Microbiota, Prebiotic, Short-chain fatty acid, Soluble corn fiber

## Abstract

Soluble corn fiber (SCF) has demonstrated prebiotic effects in clinical studies. Using an *in vitro* mucosal simulator of the human intestinal microbial ecosystem (M-SHIME^®^) model, the effects of SCF treatment on colonic microbiota composition and metabolic activity and on host-microbiome interactions were evaluated using fecal samples from healthy donors of different ages (baby [≤ 2 years], *n* = 4; adult [18–45 years], *n* = 2; elderly [70 years], *n* = 1). During the 3-week treatment period, M-SHIME^®^ systems were supplemented with SCF daily (baby, 1.5, 3, or 4.5 g/d; adult, 3 or 8.5 g/d; and elderly, 8.5 g/d). M-SHIME^®^ supernatants were evaluated for their effect on the intestinal epithelial cell barrier and inflammatory responses in lipopolysaccharide. (LPS)-stimulated cells. Additionally, short-chain fatty acid (SCFA) production and microbial community composition were assessed. In the baby and adult models, M-SHIME^®^ supernatants from SCF treated vessels protected Caco-2 membrane integrity from LPS-induced damage. SCF treatment resulted in the expansion of *Bacteroidetes*, *Firmicutes*, and *Bifidobacterial*, as well as increased SCFA production in all age groups. SCF tended to have the greatest effect on propionate production. These findings demonstrate the prebiotic potential of SCF in babies, adults, and the elderly and provide insight into the mechanisms behind the observed prebiotic effects.

**Supplementary Information:**

The online version contains supplementary material available at 10.1007/s11130-023-01043-z.

## Introduction

A healthy gut microbiome that is stable, highly diverse, and resistant to stress-related changes is important to human health [1]. Under healthy conditions, gut bacteria are involved in the metabolism of nutrients, immunomodulation, maintenance of intestinal barrier integrity, and protection from pathogenic bacteria [2]. Alterations in the gut microbiota have been linked to several gastrointestinal conditions, including inflammatory bowel disease, irritable bowel syndrome, obesity, and type 2 diabetes [3].

Dietary intake is one of several environmental factors that can influence the composition of the gut microbiome. For example, a study comparing the fecal microbiota of European children who ate a typical western diet (*i.e*., high in animal protein, sugar, starch, and fat and low in fiber), and children from an African village in Burkina Faso who ate a predominantly vegetarian diet (*i.e*., high in starch, fiber, and plant polysaccharides, and low in fat and animal protein) reported notable differences [4]. These differences included a significant increase in the *Bacteroidetes:Firmicutes* ratio in the children who ate a mostly vegetarian diet versus those who ate a European diet. Thus, dietary modulation provides a potential opportunity to modify gut microbiome composition and function.

Prebiotics are non-digestible oligosaccharides that are fermented by saccharolytic bacteria in the large intestine [5], and also provide health benefits via their effects on the intestinal microbial community. They have been defined by The International Scientific Association for Probiotics and Prebiotics as “a substrate that is selectively utilized by host microorganisms conferring a health benefit” and include dietary fibers that meet this criterion such as oligosaccharides (*e.g*., human milk oligosaccharides, fructooligosaccharides, inulin, galactooligosaccharides and resistant dextrins), polyunsaturated fatty acid, conjugated linoleic acid, phenolics, and phytochemicals [5]. Members of the gut microbiota break down prebiotics to generate energy and produce metabolites, such as short-chain fatty acids (SCFA), which are beneficial to human health [5].

Soluble corn fiber (SCF) is a highly soluble fiber that has demonstrated prebiotic effects [6]. A clinical study reported a significant increase in *Bifidobacterium *spp*.* in healthy men who consumed 21 g/d SCF for 21 days versus those who did not [7]. This change was considered to be beneficial, as it is well established that members of this bacterial genus confer positive health benefits to the human host, such as the production of beneficial metabolites (*e.g*., SCFAs and vitamins), immune system modulation, and protection against pathogens [8]. Studies have shown that SCF has good digestive tolerance [7, 9, 10], improves laxation [7, 10], has favorable blood glucose and insulin response [11], improves calcium absorption and bone calcium reabsorption [12, 13], and has potential synbiotic effects [14].

This study was conducted to evaluate the effects of SCF on host-microbiome interactions and the metabolic activity and composition of the colonic microbiota using a mucosal simulator of the human intestinal microbial ecosystem (M-SHIME®) *in vitro* model with fecal samples from healthy individual donors from different age groups (baby, adult, elderly).

## Materials and Methods

This section is presented as supplementary material.

## Results and Discussion

**Fig. 1:**
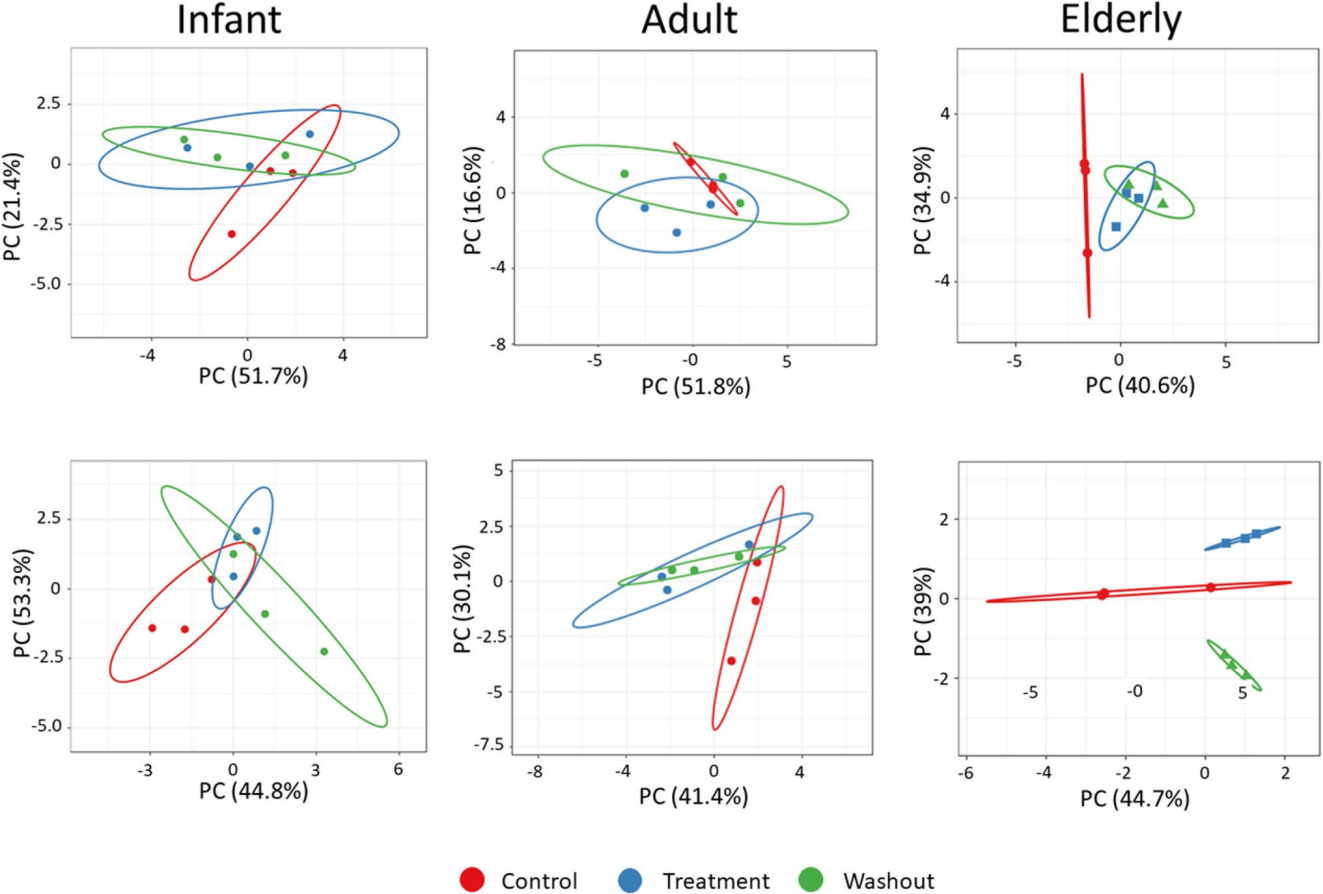
Age-dependent host-microbiota interplay in response to SCF supplementation. Principal component analysis (PCA) plots representing cell culture data (inflammatory markers [IL-1*β*, IL-6, IL-8, TNF, CXCL-10] and membrane integrity [TEER]) obtained for the control (*n* = 6 time points, red), SCF treatment (*n* = 9 time points; blue), and washout (6 time points, green) periods of the baby [AC/PC and DC; 1.5 g/d SCF, *n* = 2 (baby A and baby B); 3 g/d SCF, *n* = 1 (baby B); 4.5 g/d SCF, *n* = 1 (baby B), adult [AC and DC; 3 g/d (adult A) and 8.5 g/d SCF (adult B), *n* = 1 for each dose], and elderly (AC and DC; 8.5 g/d SCF *n* = 1) M-SHIME^®^ studies. Ellipses are drawn at the 95% confidence interval. AC, ascending colon; PC = proximal colon; DC = distal colon; IL = interleukin; M-SHIME^®^ = mucosal simulator of the human intestinal microbial ecosystem; SCF = soluble corn fiber; TEER = transepithelial electrical resistance; TNF = tumor necrosis factor. CXCL = chemokine (C-X-C motif) ligand

SCF treatment resulted in age-dependent changes to the epithelial barrier function and immune markers, suggesting a significant effect of SCF supplementation on host-microbiome interplay (Fig. 1).

**Fig. 2:**
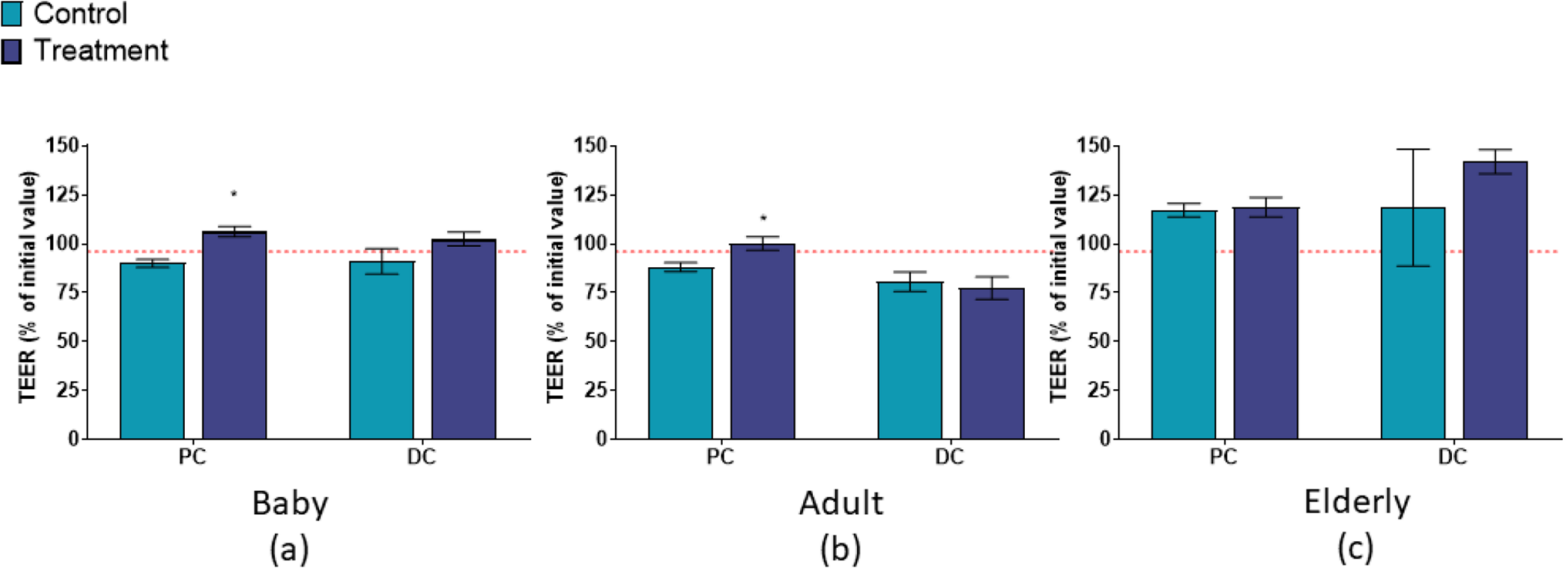
Effect of M-SHIME^®^ supernatants treated with SCF on the barrier integrity of Caco-2 cells (**a**) baby (Baby C, 3 g/d SCF), PC and DC (**b**) adult (Adult A, 3 g/d SCF), PC and DC (**c**) elderly (8.5 g/d SCF), AC and DC. Data were analyzed using a two-way ANOVA with Dunnett’s multiple comparison test (control period versus treatment period). * *p* < 0.05, red dashed line = TEER (% of initial value) for co-cultures treated with CM. Error bars represent standard error of the mean. Samples were assayed in triplicate. AC = ascending colon, ANOVA = analysis of variance, CM = Caco-2 media; DC = distal colon; M-SHIME^®^ = mucosal simulator of the human intestinal microbial ecosystem; PC = proximal colon; SCF = soluble corn fiber, TEER = transepithelial electric resistance

**Fig. 3:**
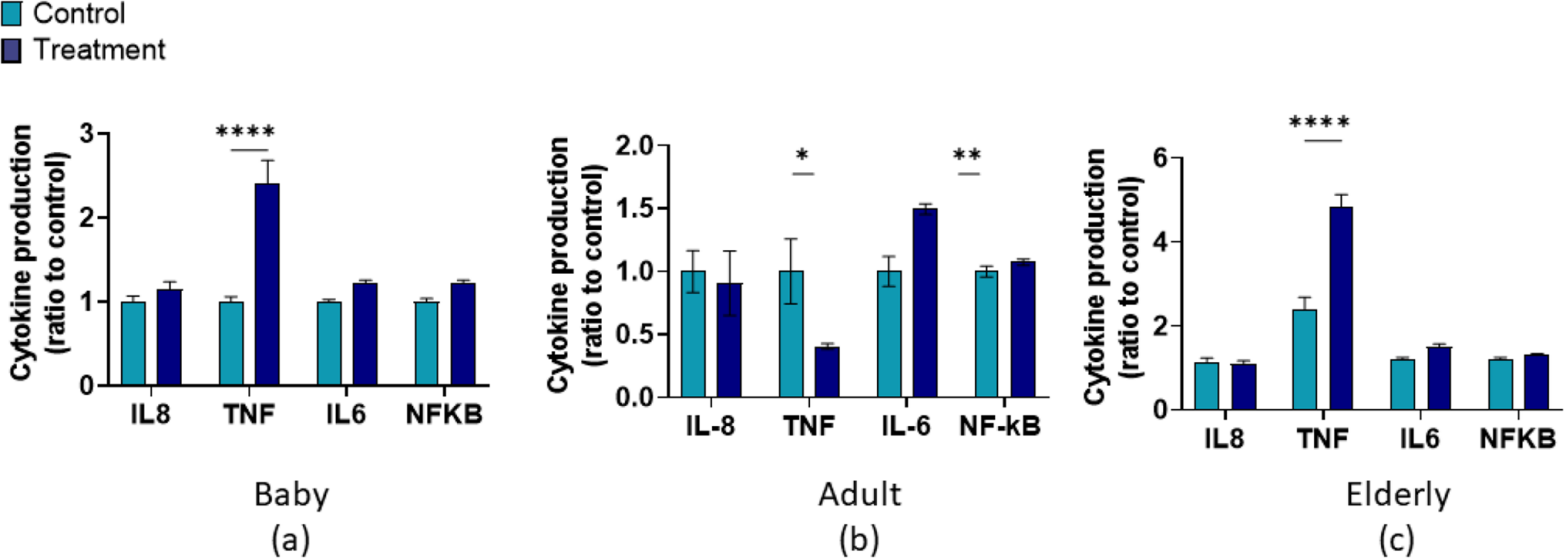
Effect of M-SHIME® PC/AC supernatants from the treatment and washout periods on cytokine and chemokine release and NFκB activity in the co-culture system for **a** Baby C (3 g/d SCF), PC **b** adult A (3 g/d SCF), PC and **c** elderly (8.5 g/d SCF), AC. Data were converted to the ratio of the control period and analyzed using a two-way ANOVA with Dunnett’s multiple comparison test (treatment period versus washout period). * *p* < 0.05, **p < 0.01, *** *p* < 0.001, red dashed line = value for co-cultures treated with supernatants from the control period. Error bars represent standard error of the mean. Samples were assayed in triplicate. AC = ascending colon, ANOVA = analysis of variance, IL = interleukin, M-SHIME® = mucosal simulator of the human intestinal microbial ecosystem; PC = proximal colon; SCF, soluble corn fiber, TNF = tumor necrosis factor

SCF treatment resulted in the protection of the Caco-2 membrane integrity in the baby and adult models, but not in the elderly model (Figs. 2 and 3). This observation could be linked to higher basal butyrate levels in the elderly donor, ~ 17–20 mmol/L, compared to adult or baby donors (~ 8–12 mmol/L). It has been previously reported that microbial-derived butyrate augments intestinal barrier function via stabilization of hypoxia-inducible factor (HIF), acting as histone deacetylase inhibitor, and affecting multiple tight junction proteins [15]. High levels of butyrate during the control period could already reinforce the epithelial barrier, protect from LPS-induced damage and mask effects of SCF supplementation.

These differences in butyrate production are likely linked to differences in dietary patterns and interpersonal microbiota background, suggesting that potential effects of SCF supplementation should be evaluated under a personalized perspective.

**Fig. 4:**
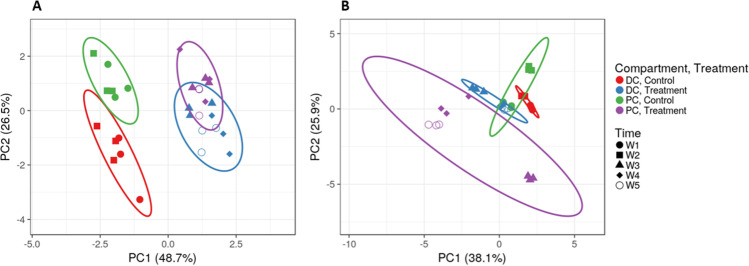
Effect of SCF on the infant microbial ecosystem of the M-SHIME^®^. **a** PCA plot including pH, gas, lactate, SCFA, branched SCFA, and ammonium levels and **b** PCA plot including qPCR levels of *Firmicutes*, *Bacteroidetes*, *Lactobacilli* and *Bifidobacteria*. Ellipses are drawn at the 95% confidence interval. Data from Baby C and Baby D (3 g/d SCF) are shown. PC = proximal colon; DC = distal colon; M-SHIME® = mucosal simulator of the human intestinal microbial ecosystem; PCA = principal component analysis; SCF = soluble corn fiber; SCFA = short-chain fatty acid. W1 = week 1 (baseline); W2 = week 2 (baseline), W3 = week 3 (SCF treatment), W4 = week 4 (SCF treatment); W5 = week 5 (SCF treatment)

In all three models, there were increases in *Bacteroidetes*, *Firmicutes*, and *Bifidobacteria* with treatment, though there was variability among the models and between donors. Treatment resulted in increased SCFA production in all three models, with the increase in propionate being most pronounced in the baby and adult models. Notably, to the authors best knowledge, this is the first report on the effects of SCF on the baby gut microbiome (Fig. 4).

**Fig. 5:**
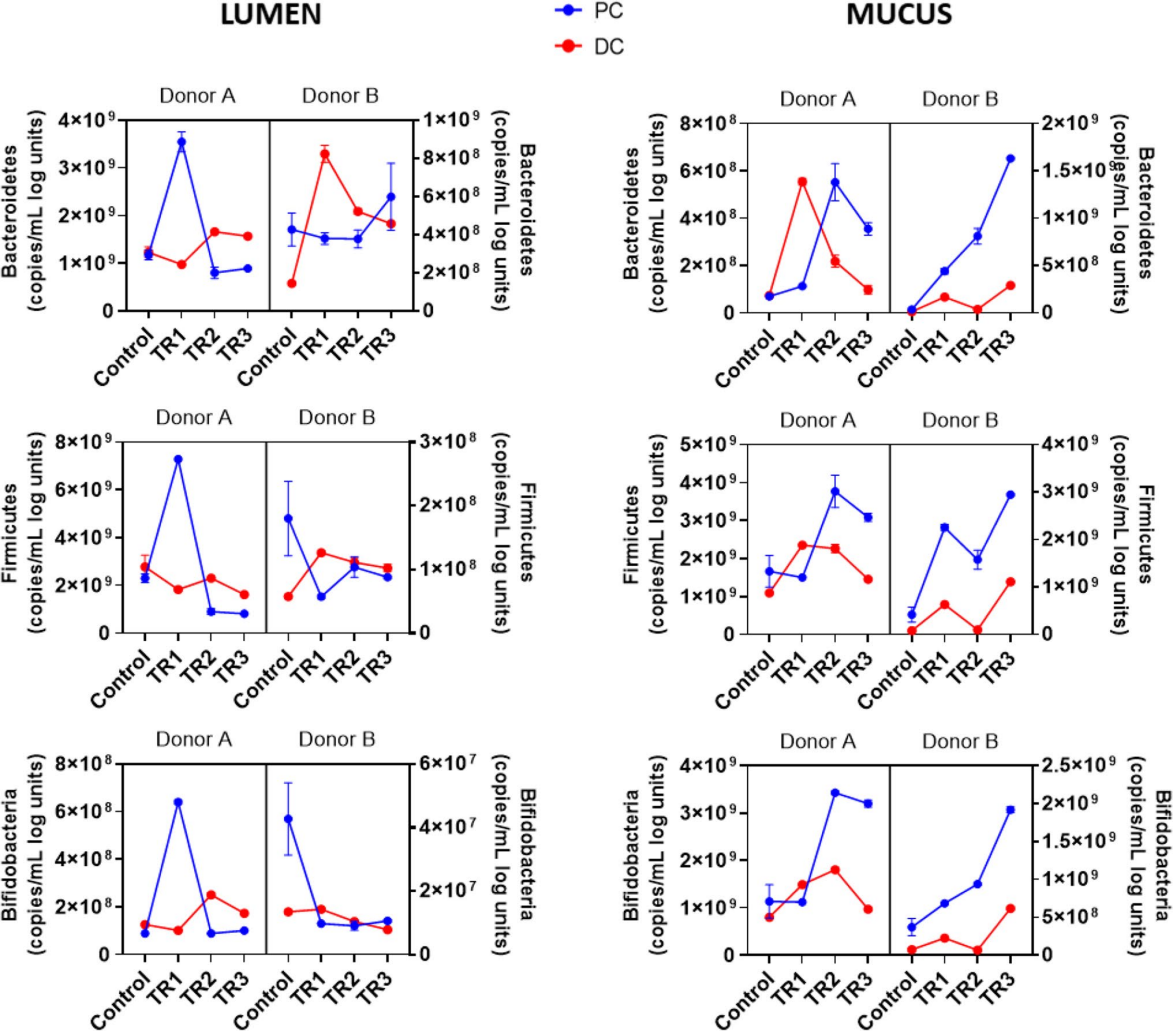
Luminal and mucosal microbial community composition during the control and treatment periods [weeks 1 (W1), 2 (W2), and 3 (W3); 3 g/d SCF] in the baby M-SHIME^®^ experiment as assessed by qPCR in Baby C (3 g/d SCF) (**b**) Baby D (3 g/d SCF). All samples were analyzed in triplicate (3 sampling points per week) and in three technical replicates of qPCR technique. Data were analyzed using a 3-way analysis of variance repeated measures model and statistically significant differences are summarized in Supplementary Table S13. PC = proximal colon; DC = distal colon; SCF = soluble corn fiber; W1 = week 1; W2 = week 2; W3 = week 3

SCF has demonstrated prebiotic effects in human studies. For example, *Bifidobacteria* numbers were increased in the feces of healthy adults after 14 or 21 days of treatment with SCF [7, 16]. An increase in *Bifidobacteria* is considered a shift towards a healthier gut microbiota [17] and is therefore expected to confer health benefits. Using the M-SHIME^®^ model, we show that SCF supports the growth of *Bifidobacteria* in the baby and elderly gut microbiota and adds to the previously published evidence of this prebiotic effect in the adult gut microbiota [7, 16]. SCF treatment also resulted in higher numbers of *Bacteroidetes* and *Firmicutes* (Fig. 5).

SCF was fermented in the colonic compartments of all three models as evidenced by the increased SCFA levels with SCF treatment. The observed SCFA increases are likely explained by the increase in *Bifidobacteria species*, which produce mainly acetate [18], *Firmicutes*, which produce mainly butyrate [19], and *Bacteroidetes*, which produce mainly succinate and acetate [20]. The succinate pathway is the most abundant route for propionate formation from hexoses and it is mostly present in the phylum *Bacteroidetes*, although some Negativicutes can form propionate from succinate as well. Therefore, the increase in *Bacteroidetes* likely explains the elevated propionate levels [19, 21].

**Fig. 6:**
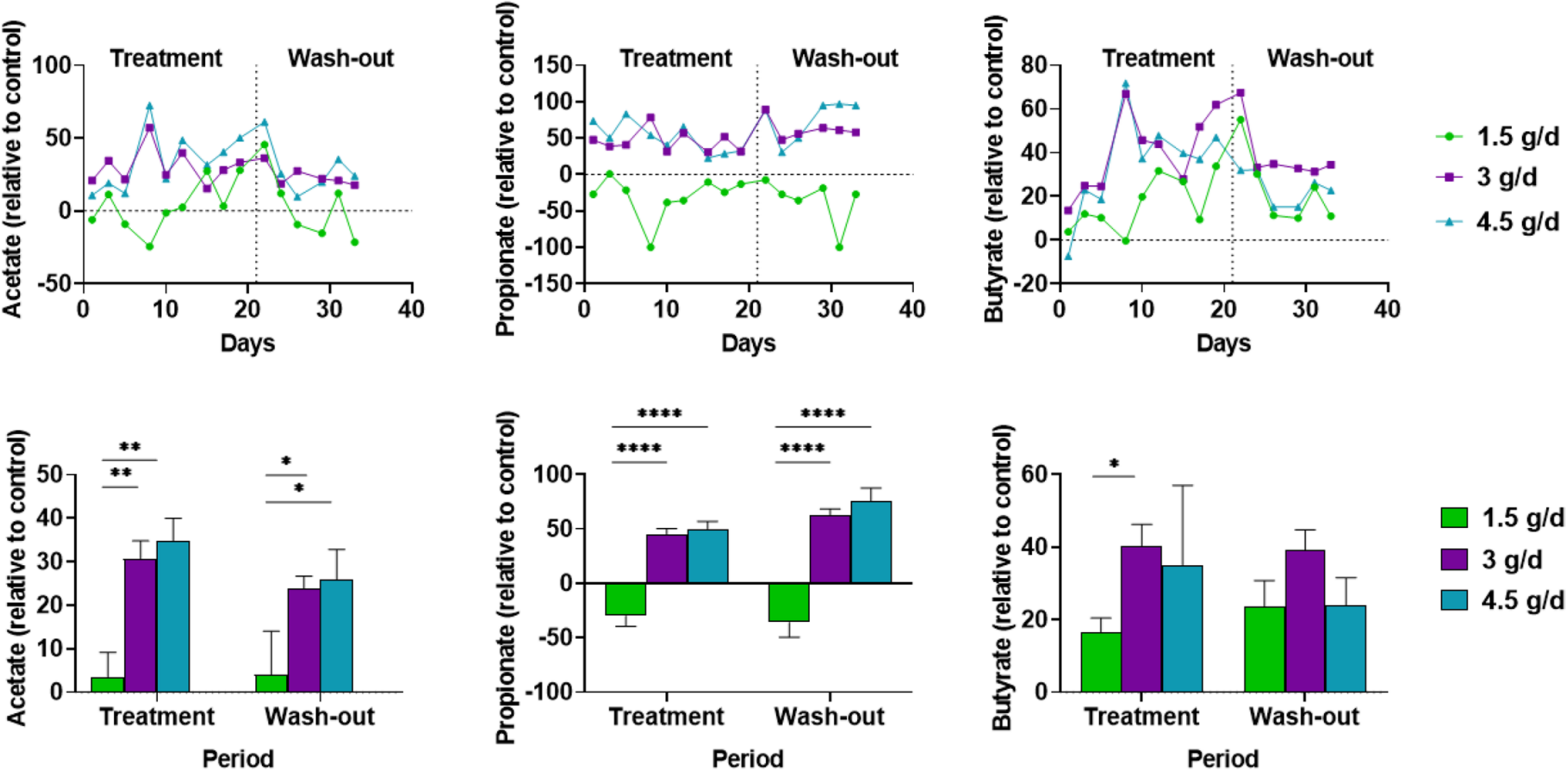
Changes in SCFA levels in the PC during the treatment (Baby B, 1.5 g/d, 3 g/d, and 4.5 g/d SCF) and washout periods in the baby M-SHIME^®^ model. The top three graphs show changes in SCFA levels over time relative to the control period. The bottom three graphs show the mean SCFA level for the treatment or washout period relative to the control period. Differences in SCFA levels between SCF doses were analyzed using a two-way ANOVA with Dunnett’s multiple comparison test. * *p* < 0.05, **p < 0.01, **** *p* < 0.0001. Samples were assayed in triplicate. ANOVA = analysis of variance; M-SHIME^®^ = mucosal simulator of the human intestinal microbial ecosystem; PC = proximal colon; SCF = soluble corn fiber; SCFA, short-chain fatty acid

SCFAs are an important energy source for intestinal epithelial cells and contribute to gut membrane barrier integrity [22]. Using *in vitro* models, butyrate and propionate have been shown to increase TEER, which indicates a role in improving gut membrane integrity [22]. The increased SCFA production observed in our study is likely responsible for the protective effect on the membrane integrity of intestinal epithelial cells observed with supernatants collected from the baby and adult models (Fig. 6, Supplementary Fig. S6 and Supplementary Table S17). Butyrate is the preferred substrate for colonocytes and is thought to promote a normal phenotype in these cells and regulate their energy metabolism [23]. Both butyrate and propionate are reported to have anti-inflammatory and anti-cancer properties [24–26] and to be involved in mitigating weight gain [24, 27]. Additionally, propionate protects from hypertensive cardiovascular damage [28], mediates resistance to Salmonella colonization [29], and lowers lipidogenesis and serum cholesterol levels [33]. A previous study evaluating a synbiotic combination of SCF and the probiotic *Lactobacillus rhamnosus GG-PB12* showed a decrease in both total cholesterol and LDL-cholesterol in elderly patients with hypercholesterolemia; however, they did not evaluate SCFA production during treatment [14]. While all three tested SCFAs increased with SCF treatment, the greatest increase was observed for propionate. Previous research has shown that SCF treatment results in increased SCFA production. In mice fed a high-fat diet, SCF supplementation significantly increased propionate and butyrate levels versus mice fed a high-fat diet alone [30]. The same study showed that SCF affected weight gain in mice fed a high-fat diet; supplementation with SCF resulted in significantly lower body weight compared with consumption of a high-fat diet alone. Additionally, SCF has been shown to significantly increase the level of acetate and propionate in the cecum of piglets [31]. The findings of the present study confirm the ability of SCF to increase SCFA production and provides a mechanism for this increase which may be explained by the changes observed in the microbial community composition with SCF treatment.

SCFAs are reported to have anti-inflammatory activity via modulation of inflammatory mediator production by macrophages [26]. Among the SCFAs, butyrate is the most active in suppressing LPS-induced TNFα and IL-6 and enhancing the production of the anti-inflammatory cytokine IL-10 [26]. IL-10 production was overall enhanced in this study, with the exception of adult B donor, supporting the previous link between fiber intake, microbiota-derived metabolites and cytokine regulation in the host. In the baby M-SHIME^®^ experiments, there was little effect overall of SCF treated supernatants on the cytokine/chemokine production or NF*κ*B activity after cells were stimulated with LPS. The only exception was TNF*α*, for which the level (relative to the control period) was significantly higher with supernatants from the washout period compared with the treatment period, indicating that something in the supernatant from washout period induced an inflammatory response. A different effect was observed in the adult study, where there was a significant increase in IL-6 and a significant decrease in TNF*α* after LPS stimulation with supernatants from the washout versus the treatment period. The increase in IL-6 and decrease in TNF*α* may indicate a delayed and continued anti-inflammatory effect after discontinuing SCF; however, this is difficult to explain as the SCFA levels declined during the washout period. IL-6 has several anti-inflammatory properties as it plays a role in resolving inflammation by promoting neutrophil clearance [32] and positively affects intestinal epithelial cell regeneration after injury [33]. There was an increase in TNF*α* (relative to supernatants from the control period) after LPS stimulation with supernatants from both the treatment and washout periods of the elderly M-SHIME^®^ study and the increase was significantly higher with supernatants from the washout period versus from the treatment period. It is possible that something in the M-SHIME^®^ supernatants induced this inflammatory response.

A study of SCF in healthy elderly participants (aged 60–80 years) reported that serum concentrations of IL-6 were decreased and that *Parabacteroides*, a member of the *Bacteroidetes* phylum, and *Ruminococcaceae*, a member of the *Firmicutes* phylum, were increased in the feces after three weeks of SCF supplementation [14]. In the present study, *in vitro* assays did not demonstrate a reduction of IL-6 after LPS stimulation when cells were exposed to SCF-treated supernatants from the elderly M-SHIME^®^. In fact, we observed an increase in TNF*α* with supernatants from the treatment, and to a greater extent, the washout period, versus the control period. It is possible that the effects of SCF are different in an *in vitro* assay system versus *in vivo*, or that this is a limitation of the fact that a single donor was used in our study, while the *in vivo* study included 40 participants. An increase in *Bacteroidetes*, *Firmicutes*, and *Bifidobacteria* with SCF treatment was observed in our elderly M-SHIME^®^ model.

This study had several limitations. First, the findings obtained using an *in vitro* model cannot be directly translated to biological responses; therefore, SCF should be further evaluated *in vivo* to determine whether the findings obtained *in vitro* translate to a biological effect. However, we do note that the SHIME^®^ model has undergone several validation studies. The fermentation profiles of pectin, xylan, arabinogalactan, and starch were confirmed to be consistent between the SHIME^®^ model and incubations with fecal microbiota from human volunteers. Additionally, bacterial metabolic phenotypes were confirmed to be preserved [34] and, using the M-SHIME^®^ model, it was confirmed that interindividual differences among human volunteers as well as unique microbial patterns for individuals were preserved *in vitro* [35]. Second, our study was conducted using the fecal microbiota of one to four donors *per* age group, thus limiting the generalizability of our findings, as donor-to-donor differences were observed in the experiments where two or more donors were tested. Finally, the baby, adult, and elderly M-SHIME^®^ studies were conducted somewhat differently, making direct comparisons of the results of the different age groups inappropriate.

## Conclusion

Although this study had some limitations, it is the first *in vitro* investigation into the effects of SCF on the baby gut microbiota. Importantly, it provides support for further studies to determine whether the prebiotic effects observed using the M-SHIME^®^ model will translate to beneficial biological effects *in vivo*. The findings using the adult M-SHIME^®^ model add to the evidence of the prebiotic effect of SCF in the adult population and provide additional insight into the changes in the microbial community activity that occur with SCF treatment. As with the adult M-SHIME^®^ studies, the effects of SCF on the elderly population reported herein expand upon previous findings and support additional studies of the prebiotic effects of SCF in this population.

## Supplementary Information

Below is the link to the electronic supplementary material.

ESM 1 (DOCX 1.70 MB)

## Data Availability

Data available upon request.
