# Supplementary material for: Age-Dependent Prebiotic Effects of Soluble Corn Fiber in M-SHIME® Gut Microbial Ecosystems
Source: Plant Foods Hum Nutr. 2023 Jan 25;78(1):213–20. doi: 10.1007/s11130-023-01043-z (PMC9947079; doi:10.1007/s11130-023-01043-z)
Supplement: Supplementary file 1 — (DOCX 1.70 MB) [file 11130_2023_1043_MOESM1_ESM.docx]

**Supplemental Materials**

**Age-dependent prebiotic effects of soluble corn fiber in M-SHIME^®^ gut microbial ecosystems**

Marta Calatayud Arroyo ^1,2#^, Ieva Laurie ^3#*^, Chloë Rotsaert ^1^, Massimo Marzorati ^1,2^, Davide Risso ^3^ and Kavita Karnik ^3^

^1^ ProDigest, Technologiepark 82, 9052 Zwijnaarde, Belgium; marta.calatayudarroyo@ugent.be (MCA), chloe.rotsaert@prodigest.eu (CR), massimo.marzorati@prodigest.eu (MM)

^2^ Center for Microbial Ecology and Technology (CMET), Faculty of Bioscience Engineering, Ghent University, Coupure Links 653, 9000 Ghent, Belgium

^3^ Tate & Lyle PLC, 5 Marble Arch, London, W1H 7EJ, United Kingdom; ieva.laurie@tateandlyle.com (IL), da-vide.risso@tateandlyle.com (DR), kavita.karnik@tateandlyle.com (KK)

* Correspondence: ieva.laurie@tateandlyle.com; Tel.: +447900632410

# Equal contribution

**Materials and Methods**

**M-SHIME^®^ model**

The M-SHIME**^®^** reactor configuration used in these studies was adapted from the originally described SHIME**^®^** model (ProDigest and Ghent University, Belgium) [1] to include simulation of both the mucus-associated and luminal microbial communities [1,2]. Details of this setup, including inoculum preparation, retention times, pH, temperature settings, mucin-covered microcosms, and reactor feed composition have been described previously [1,2]. A summary of the donors and M-SHIME**^®^** experimental details is shown in Table S1. In the M-SHIME**^®^** experiments using feces from baby donors, triple-SHIME experiments (Supplementary Fig. S1a) were run in parallel so that the dose-response effect of SCF could be measured at three doses, 1.5 g/d, 3 g/d, and 4.5 g/d (correlates with 3 g/d, 6 g/d, and 9 g/d, respectively, for a baby human). Each baby M-SHIME**^®^** unit consisted of one vessel stimulating the conditions of both the stomach and small intestine, and one each for the proximal colon (PC, pH 5.6–5.9) and distal colon (DC, pH 6.0-6.5). The M-SHIME**^®^** experiments using feces from adult or elderly donors used a standard M-SHIME**^®^** set up (Supplementary Fig. S1b), which consisted of five vessels simulating the stomach, small intestine, ascending colon (AC, pH = 5.6-5.9), transverse colon (TC, pH = 6.2–6.5), and descending colon (DC, pH 6.6–6.9) except for one adult experiment (SCF low-dose, 3 g/d) which used the same set up as the baby experiments. To simulate the gut microbiome, each of the colonic vessels was inoculated with fecal microbiota from an appropriate fecal sample originating from a single healthy baby (<1.5 years or 1.5–2 years), adult (18–45 years), or elderly (70 years) volunteer with no history of antibiotic treatment in the 6 months prior to sample collection. Adult and elderly donors followed a self-selected western diet without restrictions, and did not take probiotic or prebiotic supplementation. Baby donors were breastfed at least during the first three months of life and all consumed formula milk during the course of the experiments, combined with a standard diet as recommended by Belgian paediatricians, based on vegetables, cereals, meat and fish.

Briefly, fecal samples were collected and homogenized in anaerobic phosphate buffer (K2HPO4 8.8 g/L; KH2PO4 6.8 g/L; sodium thioglycolate 0.1 g/L; sodium dithionite 0.015 g/L) (1:5 w/v) using a stomacher bagmixer for 10 min (BagMixer 400, Interscience, Louvain-LaNeuve, Belgium). Big particles were removed by centrifugation (2 min, 500 g), and the fecal slurries 10% v/v in nutritional media [3.5 g/L K2HPO4, 10.9 g/L KH2PO4, 2 g/L NaHCO3 (Chem-lab NV, Zedelgem, Belgium), arabinogalactan (1g/L), pec-tin (2g/L), xylan (2g/L), 2 g/L Yeast Extract, 2 g/L peptone (Oxoid, Aalst, Belgium), 0.5 g/L L-cysteine and 2 mL/L Tween80 (Sigma-Aldrich, Bornem, Belgium)] were used as inoculum for the colonic incubations. Samples were obtained followed by ethical approval of the University Hospital Ghent, reference number B670201836585. Colonic vessels in all M-SHIME**^®^** experiments had a mucosal environment consisting of 80 mucin agar-covered microcosms (AnoxKaldnes K1 carrier; AnoxKaldnes AB, Lund, Sweden), placed in a polyethylene netting (Zakkencentrale, Rotterdam, The Netherlands). Mucin-agar was prepared by boiling autoclaved distilled H2O containing 5% porcine mucin type II and 1% agar (Sigma-Aldrich, Belgium). After fecal inoculation of colonic vessels, a static incubation of 16h was performed to stabilize the system. Subsequently, a semi-continuous mode was started, with the peristaltic pumps supplying each colon vessel with 200 mL of pre-digested feed three times per day every 8 h. Pre-digestion consisted of a sequential mixture of 140 mL of nutritional media (45 min incubation in the stomach compartment, pH 2) and 60 mL of pancreatic juice (NaHCO3 12.5 g/L, bile salts 6.0 g/L and pancreatin 0.9 g/L; Sigma-Aldrich, Belgium).

From starting the semi-continuous mode, two-week startup period was run to allow the microbial community to be established and differentiated according to local environmental conditions. This was followed by a two-week control period without any treatment; samples collected during this period allowed for the determination of the baseline microbial community activity and supplemented with soluble corn fiber daily (baby, 1.5 g/d, 3 g/d, 4.5 g/d; adult, 3 g/d, 8.5 g/d [correlates with 6 g/d and 17 g/d, respectively, for an adult human]; elderly, 8.5 g/d [correlates with 17 g/d for an adult human]). Some experiments also included a two-week washout period, during which the reactors were fed with standard feed (no SCF), determining whether microbial parameters returned to baseline or if permanent changes could be observed. Soluble corn fiber (PROMITOR® Soluble Corn Fiber, SCF) was provided by Tate & Lyle PLC, UK, as dry powder. Fig. S2 shows a schematic representation of the experimental setup used in this research.

**Table S1.** Summary of donor and experimental details

| **Donor** | **Sex** | **SCF dose**  **(g/d)** | **M-SHIME^®^ configuration** |
| --- | --- | --- | --- |
| Elderly | Male | 8.5 | AC/TC/DC |
| Adult A | Male | 3 | PC/DC |
| Adult B | Male | 8.5 | AC/TC/DC |
| Baby A (1.5 years) | Female | 1.5 | AC/TC/DC |
| Baby B (< 1.5 years) | Male | 1.5 | PC/DC |
| Baby B (<1.5 years) | Male | 3 | PC/DC |
| Baby B (<1.5 years) | Male | 4.5 | PC/DC |
| Baby C (2 years) | Male | 3 | PC/DC |
| Baby D (2 years) | Female | 3 | PC/DC |

SCF = soluble corn fiber.

**
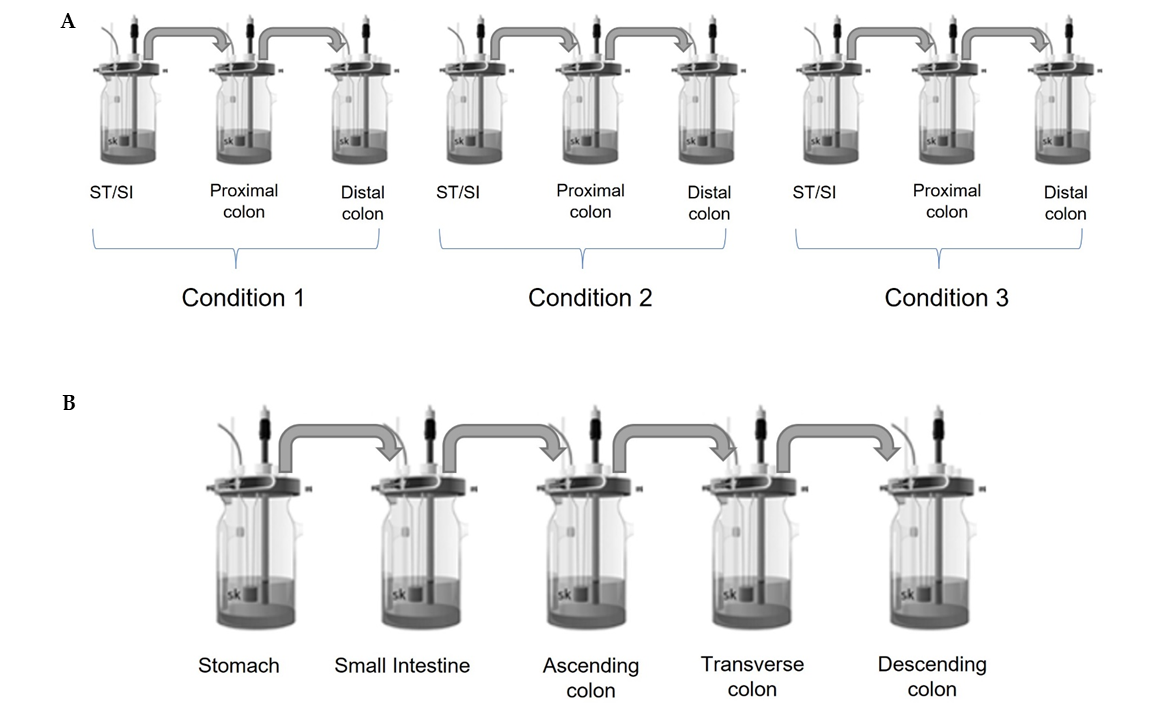
**

Fig. S1 M-SHIME^®^ reactor setups used in this study (A) triple M-SHIME^®^ and (B) standard M-SHIME^®^. M-SHIME^®^ = mucosal simulator of the human intestinal microbial ecosystem


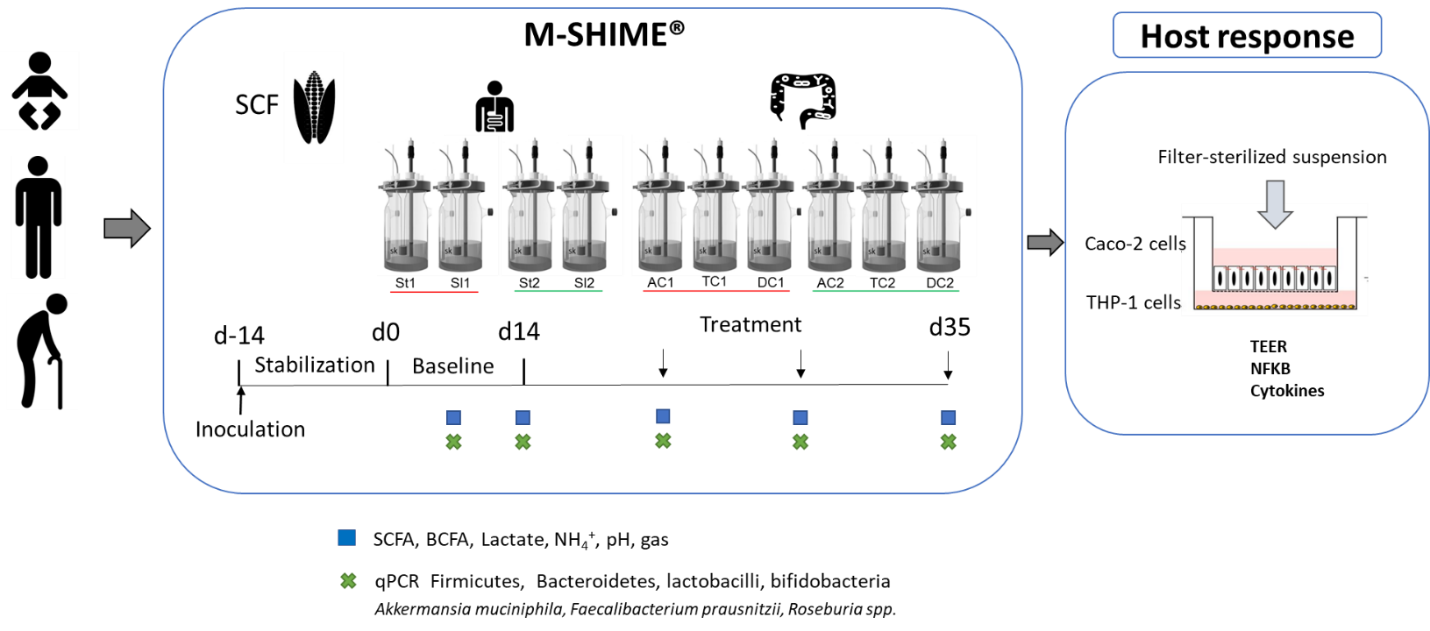


**Fig. S2** Schematic representation of the experimental setup used in this research. SCF = soluble corn fiber; M-SHIME = mucosal simulator of the human gut microbial ecosystem; TEER = transepithelial electrical resistance; d = day; St = stomach reactor; SI = small intestinal reactor; AC = ascending colon; TC = transverse colon; DC = distal colon

**Host response**

Co-culture experiments using Caco-2 (HTB-37, American Type Culture Collection; passage number 50-55; 7.5x104 cells/cm2) and THP1-Blue™ cells (InvivoGen, San Diego, CA, USA; passage number 70-75, 4x104 cells/cm2) were performed as described previously [3,4]. Briefly, co-cultures were exposed to sterile-filtered (0.22 µM) colonic M-SHIME**^®^** suspensions for 24h, after which the transepithelial electrical resistance (TEER) of Caco-2 cells was measured for each well. Cells were then stimulated with 500 ng/mL ultrapure lipopolysaccharide (LPS; Escherichia coli K12, InvivoGen) added to the basolateral compartment and after 6h, basal supernatants were collected and processed for the measurement of cytokines, chemokines, and NFκB activity. Co-culture experiments were performed in technical triplicates. To determine NFκB activity, SEAP levels in the co-culture basolateral supernatants were measured using the QUANTI-Blue reagent (InvivoGen) according to the manufacturer's instructions. Levels of human IL-8, TNFα, IL-6, IL-1β, IL-10, and CXCL10 in the co-culture basolateral supernatants were measured using Luminex® multiplex (Affymetrix-eBioscience, Waltham, MA, USA). Assays were performed according to the manufacturer's instructions.

**Microbial community activity and composition**

SCFA (acetate, propionate, butyrate) levels were monitored three times per week as described previously [5]. Briefly, SCFA were extracted from the samples with diethyl ether, after the addition of 2-methyl hexanoic acid as an internal standard. Extracts were analysed using a GC-2014 gas chromatograph (Shimadzu, 's-Hertogenbosch, the Netherlands), equipped with a capillary fatty acid-free EC-1000 Econo-Cap column (dimensions: 25 mm × 0.53 mm, film thickness 1.2 μM; Alltech, Laarne, Belgium), a flame ionization detector and a split injector. The injection volume was 1 μL and the temperature profile was set from 110 to 160 °C, with a temperature increase of 6 °C min−1. The carrier gas was nitrogen, and the temperature of the injector and detector were 100 and 220 °C, respectively.

Before (day 14) and after treatment with SCF (weekly on days 16, 26, and 35), the microbial community composition was sampled and assessed. The DNA was extracted from a pellet of bacterial cells originated from either 1 mL luminal samples or 0.1 g mucus samples after centrifugation for 5 min at 7700g. A Fastprep-24 device (MP BioMedicals, Illkirch, France) was used for homogenization (two cycles of 40 s at 4 m/s) [6]. Quality control PCR was conducted using Taq DNA Polymerase with the Fermentas PCR Kit according to the manufacturers’ instructions (Thermo Fisher Scientific, Waltham, U.S.A.). The DNA quality was verified by electrophoresis on a 2% (w/v) agarose gel for 30 min at 100 V. qPCR assays were performed using a StepOnePlus Real-Time PCR system (Applied Biosystems, Foster City, CA), using the primers and conditions described in Table S2 and S3. Each sample was analyzed in technical triplicates and outliers (more than 1 cycle threshold (CT) difference) were omitted. Melt curve peaks were checked for each sample and primer set. The efficiency of standard curves varied between 91% and 107%. Results are reported as log(16S rRNA gene copies/mL).

**Table S2.** Primers used to quantify Bifidobacteria, Firmicutes, Bacteroidetes and Lactobacillus populations by qPCR.

| **Targeted group** | **Primer name** | **Sequence (5’ – 3’)** | **Reference** |
| --- | --- | --- | --- |
| **Total bacteria** | **BacTF** | TCCTACGGGAGGCAGCAGT | [7] |
|  | **BacTR** | GGACTACCAGGGTATCTAATCCTGTT |  |
| ***Bifidobacterium* spp.** | Bif243F | TCGCGTCYGGTGTGAAAG | [8] |
|  | Bif243R | CCACATCCAGCRTCCAC |  |
| **Firmicutes (phylum)** | Firm934F | GGAGYATGTGGTTTAATTCGAAGCA | [9] |
|  | Firm1060R | AGCTGACGACAACCATGCAC |  |
| **Bacteroidetes (phylum)** | Bact934F | GGARCATGTGGTTTAATTCGATGAT |  |
|  | Bact1060R | AGCTGACGACAACCATGCAG |  |
| ***Lactobacillus* spp.** | F_lacto_05 | AGCAGTAGGGAATCTTCCA | [10] |
|  | R_lacto_04 | CGCCACTGGTGTTCYTCCATATA |  |

**Table S3.** Conditions used to quantify total bacteria, bifidobacteria, Firmicutes, Bacteroidetes and lactobacillus populations by qPCR.

| Program | Cycle | Temperature (°C) | Heating (hh:mm:ss) | Ramp (°C/s) |
| --- | --- | --- | --- | --- |
| Pre-incubation | 1 | 95 | 00:10:00 | 1.6 |
| Amplification | 40 | 95 | 00:00:15 | 1.6 |
|  |  | 60 | 00:00:30 | 1.55 |
|  |  | 72 | 00:00:30 | 1.6 |
| Melting curves | 1 | 95 | 00:00:15 | 1.6 |
|  |  | 60 | 00:01:00 | 1.55 |
|  |  | 75 | 00:00:15 | 0.075 |

**Statistical methods**

All statistical analyses were performed using GraphPad Prism version 9 for Windows (GraphPad Software, San Diego, U.S.A.). A p value of <0.05 was considered significant. Principal Component Analysis (PCA) was per-formed using ClustVis (https://biit.cs.ut.ee/clustvis/) after standardizing the data [11]. Comparisons of the data were analyzed using a two-way ANOVA.

**Results and Discussion**

Table S4. Effect of M-SHIME supernatants from baby A on epithelial barrier function, cytokine production and NFΚβ activity of the Caco-2 model. Data represent mean ± standard deviation (n=3) of cells exposed to M-SHIME supernatants from week 2 of control period, week 3 of treatment period (1.5 g/L) and week 2 of washout period. TEER values measured after 6 hours of LPS proinflammatory trigger in the presence of M-SHIME supernatants are refereed to baseline values obtained after exposure to M-SHIME supernatants (24 hours), before LPS addition. Statistically significant differences (ordinary one-way ANOVA) between control period and treatment or washout periods are marked in bold. Differences between treatment period and washout are marked with italics.

|  |  | TEER | | | LY | | | IL-8 | | | TNF | | | IL-6 | | | IL-10 | | | NF-kB | | |
| --- | --- | --- | --- | --- | --- | --- | --- | --- | --- | --- | --- | --- | --- | --- | --- | --- | --- | --- | --- | --- | --- | --- |
|  |  | % to baseline | | | µM | | | ng/mL | | | | | | pg/mL | | | | | | OD630 | | |
| Control | AC | 77.53 | ± | 9.90 | 7.23 | ± | 0.98 | 13.604 | ± | 1.617 | 6.077 | ± | 2.416 | 55.026 | ± | 4.320 | 33.540 | ± | 3.625 | 0.785 | ± | 0.038 |
| Treatment |  | **94.82** | **±** | **5.78** | 5.26 | ± | 1.63 | **11.093** | **±** | **1.364** | 7.401 | ± | 3.299 | 44.477 | ± | 0.774 | **18.698** | **±** | **3.800** | 0.722 | ± | 0.030 |
| Washout |  | **92.74** | **±** | **3.24** | ***3.25*** | ***±*** | ***0.43*** | 11.585 | ± | 1.496 | 4.391 | ± | 5.428 | 49.810 | ± | 16.814 | 22.195 | ± | 8.135 | 0.793 | ± | 0.046 |
| Control | TC | 68.73 | ± | 5.67 | 8.76 | ± | 0.07 | 17.504 | ± | 0.745 | 6.541 | ± | 3.116 | 60.836 | ± | 6.335 | 52.428 | ± | 9.705 | 0.866 | ± | 0.074 |
| Treatment |  | **83.39** | **±** | **7.38** | **6.07** | **±** | **0.94** | **13.337** | **±** | **1.102** | 2.449 | ± | 1.467 | 49.965 | ± | 2.096 | **26.476** | **±** | **2.133** | **0.681** | **±** | **0.055** |
| Washout |  | **86.98** | **±** | **3.72** | **4.45** | **±** | **0.56** | **14.104** | **±** | **0.082** | 6.462 | ± | 3.192 | *64.354* | *±* | *6.237* | ***41.960*** | ***±*** | ***10.445*** | 0.760 | ± | 0.074 |
| Control | DC | 72.67 | ± | 9.05 | 7.45 | ± | 0.74 | 15.036 | ± | 0.197 | 3.787 | ± | 0.423 | 57.661 | ± | 5.142 | 37.108 | ± | 4.439 | 0.780 | ± | 0.079 |
| Treatment |  | **85.57** | **±** | **6.24** | 7.14 | ± | 1.33 | 13.454 | ± | 2.582 | 6.763 | ± | 1.422 | 50.366 | ± | 7.960 | 31.204 | ± | 7.969 | 0.713 | ± | 0.053 |
| Washout |  | 75.84 | ± | 2.76 | 6.44 | ± | 1.44 | 14.589 | ± | 0.195 | 4.486 | ± | 2.369 | 59.428 | ± | 5.632 | *46.499* | *±* | *5.813* | 0.808 | ± | 0.045 |

Table S5. Effect of M-SHIME supernatants from Baby B on epithelial barrier function of the Caco-2 model. Data represent mean ± standard deviation (n=3) of cells exposed to M-SHIME supernatants from week 2 of control period, week 3 of treatment period (1.5 g/L, 3 g/L ad 4.5 g/L) and week 2 of washout period, in fecal simulations from donor Baby B. Epithelial barrier was evaluated measuring transepithelial electrical resistance (TEER) and transport to the basolateral compartment of the fluorescent marker Lucifer yellow (LY). TEER values measured after 6 hours of LPS proinflammatory trigger in presence of M-SHIME supernatants are refereed to baseline values obtained after exposure to M-SHIME supernatants (24 hours), before LPS addition. Statistically significant differences (ordinary one-way ANOVA) between control period and treatment or washout periods are marked in bold. Differences between treatment period and washout are marked with italics.

|  | Proximal colon | | | | | | | | | | | | | | | | | | |
| --- | --- | --- | --- | --- | --- | --- | --- | --- | --- | --- | --- | --- | --- | --- | --- | --- | --- | --- | --- |
|  | TEER (%) | | | | | | | | | LY (µM) | | | | | | | | | |
|  | SCF 1.5 g/L | | | SCF 3 g/L | | | SCF 4.5 g/L | | | SCF 1.5 g/L | | | SCF 3 g/L | | | | SCF 4.5 g/L | | |
| Control | 110.55 | ± | 2.44 | 118.99 | ± | 2.79 | 109.14 | ± | 6.80 | 3.83 | ± | 0.52 | 2.75 | ± | 0.24 | 3.12 | | ± | 0.76 |
| Treatment | **97.83** | **±** | **2.75** | **98.55** | **±** | **4.59** | 103.94 | ± | 0.51 | **4.49** | **±** | **0.30** | **4.23** | **±** | **0.32** | 3.73 | | ± | 0.05 |
| Wash-out | **102.83** | **±** | **4.01** | ***107.87*** | ***±*** | ***6.02*** | ***119.79*** | ***±*** | ***2.59*** | *3.34* | *±* | *0.20* | ***6.21*** | ***±*** | ***0.67*** | ***5.31*** | | ***±*** | ***0.13*** |
|  |  |  |  |  |  |  |  |  |  |  |  |  |  |  |  |  | |  |  |
|  | Distal colon | | | | | | | | | | | | | | | | | | |
|  | TEER (%) | | | | | | | | | LY (µM) | | | | | | | | | |
| Control | 107.73 | ± | 3.07 | 108.89 | ± | 0.82 | 107.67 | ± | 3.89 | 3.31 | ± | 0.32 | 3.61 | ± | 0.83 | 3.13 | | ± | 0.60 |
| Treatment | **100.57** | **±** | **4.34** | **97.31** | **±** | **4.45** | **100.85** | **±** | **2.91** | **4.03** | **±** | **0.36** | 4.18 | ± | 0.80 | 3.90 | | ± | 0.45 |
| Wash-out | **100.16** | **±** | **2.49** | *108.71* | *±* | *8.09* | ***117.07*** | ***±*** | ***0.79*** | 3.46 | ± | 0.42 | ***5.31*** | ***±*** | ***0.20*** | ***6.42*** | | ***±*** | ***1.27*** |

Table S6. Effect of M-SHIME supernatants from Baby B on cytokine production and NFΚβ activity of the Caco-2 model. Data represent mean ± standard deviation (n=3) of cells exposed to M-SHIME supernatants from proximal and distal colon at week 2 of control period, week 3 of treatment period (1.5 g/L, 3 g/L ad 4.5 g/L) and week 2 of washout period. Statistically significant differences (ordinary one-way ANOVA) between control period and treatment or washout periods are marked in bold. Differences between treatment period and washout are marked with italics. PC = proximal colon; DC = distal colon.

| SCF dose | Compartment | Treatment period | IL-8 | | | TNF | | | IL-6 | | | IL10 | | | NF-kB activity | | |
| --- | --- | --- | --- | --- | --- | --- | --- | --- | --- | --- | --- | --- | --- | --- | --- | --- | --- |
|  |  |  | ng/mL | | | | | | pg/mL | | | | | | OD630 | | |
| 1.5 g/d | PC | Control | 24.32 | ± | 0.89 | 20.73 | ± | 3.80 | 87.49 | ± | 5.08 | 93.93 | ± | 4.86 | 0.79 | ± | 0.01 |
|  |  | Treatment | 22.25 | ± | 1.11 | 21.55 | ± | 3.27 | 89.60 | ± | 4.05 | 90.33 | ± | 5.62 | 0.79 | ± | 0.02 |
|  |  | Wash-out | 24.65 | ± | 2.40 | 17.23 | ± | 3.26 | 80.79 | ± | 2.60 | 97.02 | ± | 2.78 | ***0.82*** | ***±*** | ***0.01*** |
|  | DC | Control | 19.92 | ± | 2.09 | 18.45 | ± | 1.13 | 109.75 | ± | 1.31 | 189.33 | ± | 9.82 | 0.87 | ± | 0.01 |
|  |  | Treatment | **25.64** | **±** | **0.77** | 15.25 | ± | 3.27 | 133.37 | ± | 9.37 | 182.97 | ± | 8.27 | **0.92** | **±** | **0.01** |
|  |  | Wash-out | 23.08 | ± | 4.09 | 16.33 | ± | 3.70 | 135.42 | ± | 6.38 | ***207.69*** | ***±*** | ***2.54*** | **0.92** | **±** | **0.02** |
| 3 g/d | PC | Control | 27.48 | ± | 2.64 | 22.16 | ± | 3.37 | 112.83 | ± | 7.51 | 164.28 | ± | 3.08 | 0.82 | ± | 0.01 |
|  |  | Treatment | 26.19 | ± | 1.68 | **12.42** | **±** | **3.69** | **126.78** | **±** | **2.98** | **186.69** | **±** | **2.35** | **0.87** | **±** | **0.01** |
|  |  | Wash-out | 26.50 | ± | 4.25 | 17.99 | ± | 5.19 | 121.41 | ± | 6.60 | ***146.93*** | ***±*** | ***7.12*** | ***0.95*** | ***±*** | ***0.03*** |
|  | DC | Control | 24.67 | ± | 2.76 | 19.57 | ± | 3.89 | 120.17 | ± | 12.68 | 182.04 | ± | 9.83 | 0.84 | ± | 0.01 |
|  |  | Treatment | 24.11 | ± | 3.16 | 13.33 | ± | 4.55 | **136.70** | **±** | **3.44** | **210.64** | **±** | **6.25** | **0.89** | **±** | **0.02** |
|  |  | Wash-out | 25.04 | ± | 3.14 | 19.19 | ± | 5.24 | 130.57 | ± | 9.37 | ***196.09*** | ***±*** | ***6.01*** | ***0.93*** | ***±*** | ***0.03*** |
| 4.5 g/d | PC | Control | 23.98 | ± | 3.44 | 18.40 | ± | 1.91 | 99.09 | ± | 7.04 | 116.25 | ± | 5.71 | 0.83 | ± | 0.02 |
|  |  | Treatment | 24.72 | ± | 2.27 | 18.55 | ± | 1.88 | 108.14 | ± | 5.36 | **143.32** | **±** | **7.55** | 0.83 | ± | 0.01 |
|  |  | Wash-out | 25.64 | ± | 4.62 | ***14.00*** | ***±*** | ***2.30*** | 99.88 | ± | 1.53 | **130.24** | **±** | **4.01** | ***0.88*** | ***±*** | ***0.02*** |
|  | DC | Control | 22.47 | ± | 2.78 | 15.07 | ± | 2.52 | 129.76 | ± | 5.31 | 200.40 | ± | 11.73 | 0.90 | ± | 0.01 |
|  |  | Treatment | 25.81 | ± | 2.19 | **9.89** | **±** | **1.24** | 136.58 | ± | 7.24 | 208.56 | ± | 10.60 | **0.93** | **±** | **0.02** |
|  |  | Wash-out | 26.23 | ± | 2.93 | ***25.09*** | ***±*** | ***0.81*** | 134.81 | ± | 9.92 | 205.28 | ± | 4.29 | ***0.97*** | ***±*** | ***0.01*** |

Table S7. Effect of M-SHIME supernatants from baby D on epithelial barrier function assessed by TEER. Data represent mean ± standard deviation (n=3) of cells exposed to M-SHIME supernatants from week 2 of control period, week 3 of treatment period (1.5 g/L). TEER values measured after 6 hours of LPS proinflammatory trigger in presence of M-SHIME supernatants are refereed to baseline values obtained after exposure to M-SHIME supernatants (24 hours), before LPS addition. PC = proximal colon; DC = distal colon; SCF = soluble corn fiber. No significant differences between control condition in each compartment and SCF treatment were detected.

| **Compartment** | **Treatment** | **TEER (% initial value)** | | |
| --- | --- | --- | --- | --- |
| PC | Control | 109.17 | ± | 6.08 |
|  | SCF 3 g/L | 115.48 | ± | 2.74 |
| DC | Control | 86.54 | ± | 5.55 |
|  | SCF 3 g/L | 94.04 | ± | 6.27 |

Table S8. Effect of M-SHIME supernatants from baby D on cytokine production and NFΚβ activity of the Caco-2 model. Data represent mean ± standard deviation (n=3) of cells exposed to M-SHIME supernatants from week 2 of control period, week 3 of treatment period (3 g/L SCF). Statistically significant differences (ordinary one-way ANOVA) between control period and treatment or washout periods are marked in bold. Differences between treatment period and washout are marked with italics. PC = proximal colon; DC = distal colon; LPS = lipopolysaccharide from *E. coli;* SCF = soluble corn fiber.

| Condition | Treat-  ment | IL-8 | | | TNF-a | | | IL-10 | | | IL-1b | | | IL-6 | | | NF-kB activity | | |
| --- | --- | --- | --- | --- | --- | --- | --- | --- | --- | --- | --- | --- | --- | --- | --- | --- | --- | --- | --- |
|  |  | pg/mL | | | | | | | | | | | | | | | OD630 | | |
|  |  | PROXIMAL COLON | | | | | | | | | | | | | | | | | |
| LPS- |  | 27770.2 | ± | 1357.6 | 72.4 | ± | 10.6 | 1.2 | ± | 0.01 | 28.7 | ± | 7.7 | 13.7 | ± | 23.7 | 0.3 | ± | 0.01 |
| LPS+ |  | 67328.8 | ± | 7862.5 | 2975.5 | ± | 2754.9 | 5.2 | ± | 0.2 | 130.8 | ± | 2.0 | 256.6 | ± | 56.3 | 0.6 | ± | 0.1 |
| SCF (+LPS) | Control | 52877.7 | ± | 4823.5 | 3475.6 | ± | 1334.7 | 11.1 | ± | 0.5 | 271.7 | ± | 7.9 | 365.2 | ± | 21.9 | 0.7 | ± | 0.01 |
|  | SCF | 68265.1 | ± | 14178.4 | 3016.9 | ± | 177.3 | **16.1** | **±** | **0.5** | 255.2 | ± | 26.7 | **471.6** | **±** | **19.2** | 0.8 | ± | 0.1 |
|  |  | DISTAL COLON | | | | | | | | | | | | | | | | | |
| LPS- |  | 40567.0 | ± | 3932.0 | 58.8 | ± | 2.2 | 2.2 | ± | 0.2 | 30.2 | ± | 10.4 | 0.0 | ± | 0.0 | 0.4 | ± | 0.01 |
| LPS+ |  | 68719.7 | ± | 7593.9 | 3086.2 | ± | 1724.1 | 4.2 | ± | 0.1 | 83.5 | ± | 7.9 | 213.8 | ± | 48.7 | 0.6 | ± | 0.02 |
| SCF (+LPS) | Control | 63203.1 | ± | 7347.4 | 4291.5 | ± | 1621.4 | 12.1 | ± | 2.0 | 236.2 | ± | 26.4 | 558.1 | ± | 41.1 | 0.7 | ± | 0.1 |
|  | SCF | 73458.7 | ± | 27360.2 | 1689.4 | ± | 1334.6 | 13.7 | ± | 3.5 | **194.6** | **±** | **20.4** | **689.5** | **±** | **48.2** | **0.9** | **±** | **0.2** |

Table S9. Effect of M-SHIME supernatants from adult B on epithelial barrier function, cytokine production and NFkb activity of the Caco-2 model. Data represent mean ± standard deviation (n=3) of cells exposed to M-SHIME supernatants from week 2 of control period, week 3 of treatment period (8.5 g/L) and week 2 of washout period. TEER values measured after 6 hours of LPS proinflammatory trigger in presence of M-SHIME supernatants are refereed to baseline values obtained after exposure to M-SHIME supernatants (24 hours), before LPS addition. Statistically significant differences (ordinary one-way ANOVA) between control period and treatment or washout periods are marked in bold. Differences between treatment period and washout are marked with italics. TEER = transepithelial electrical resistance; PC = proximal colon; DC = distal colon; LPS = lipopolysaccharide from *E. coli;* SCF = soluble corn fiber

| Condition | Compartment | IL8 | | | TNF | | | IL10 | | | IL6 | | | NF-Kb | | | TEER | | | LY | | |
| --- | --- | --- | --- | --- | --- | --- | --- | --- | --- | --- | --- | --- | --- | --- | --- | --- | --- | --- | --- | --- | --- | --- |
|  |  | ng/mL | | | | | | pg/mL | | | | | | OD630 | | | % initial value | | | uM | | |
| Control | AC | 28.73 | ± | 1.74 | 11.74 | ± | 5.38 | 65.62 | ± | 9.33 | 136.12 | ± | 3.06 | 1.23 | ± | 0.03 | 112.07 | ± | 2.20 | 3.95 | ± | 0.29 |
| Treatment |  | 31.06 | ± | 4.03 | **3.59** | **±** | **1.47** | **85.14** | **±** | **6.97** | **200.13** | **±** | **23.49** | 1.36 | ± | 0.10 | 108.19 | ± | 17.16 | 3.73 | ± | 0.21 |
| Washout |  | n.a | | | n.a | | | n.a | | | n.a | | | n.a | | | 98.50 | ± | 7.41 | ***7.06*** | ***±*** | ***2.67*** |
| Control | TC | 29.53 | ± | 4.41 | 12.31 | ± | 2.90 | 83.74 | ± | 7.61 | 154.31 | ± | 8.21 | 1.18 | ± | 0.02 | 117.91 | ± | 1.14 | 3.48 | ± | 0.48 |
| Treatment |  | 29.02 | ± | 0.11 | **6.11** | **±** | **1.94** | **67.07** | **±** | **4.09** | 165.51 | ± | 11.15 | 1.25 | ± | 0.03 | 118.79 | ± | 2.60 | **5.71** | **±** | **0.07** |
| Washout |  | *35.42* | *±* | *4.41* | *14.98* | *±* | *0.38* | ***11.50*** | ***±*** | ***0.68*** | ***41.36*** | ***±*** | ***16.00*** | ***0.81*** | ***±*** | ***0.20*** | 109.62 | ± | 11.24 | 4.84 | ± | 0.01 |
| Control | DC | 31.25 | ± | 5.61 | 10.76 | ± | 2.08 | 87.98 | ± | 15.04 | 155.92 | ± | 14.47 | 1.37 | ± | 0.07 | 109.74 | ± | 8.04 | 2.87 | ± | 0.10 |
| Treatment |  | 33.45 | ± | 1.40 | 4.72 | ± | 0.49 | 79.21 | ± | 4.11 | 144.53 | ± | 2.39 | 1.34 | ± | 0.07 | 102.15 | ± | 3.20 | 4.24 | ± | 0.63 |
| Washout |  | 32.56 | ± | 6.04 | 9.36 | ± | 6.42 | ***12.48*** | ***±*** | ***4.27*** | ***60.05*** | ***±*** | ***6.27*** | ***1.05*** | ***±*** | ***0.05*** | 104.95 | ± | 2.69 | 4.28 | ± | 1.34 |

Table S10 Microbial community composition in the proximal and distal colon of the baby M-SHIME**^®^** experiment (donor baby A) during the control, treatment and washout periods (1.5 g/d SCF) as assessed by qPCR in the luminal and mucosal compartments. Data represent mean ± standard deviation of the mean (n=3 time points per period and n=3 technical replicates per sample). PC = proximal colon; DC = distal colon. M-SHIME = mucosal simulator of the human intestinal microbial ecosystem

|  |  | **Total bacteria** | | | **Bacteroidetes group** | | | **Firmicutes group** | | | **Lactobacilli** | | | **Bifidobacteria** | | |
| --- | --- | --- | --- | --- | --- | --- | --- | --- | --- | --- | --- | --- | --- | --- | --- | --- |
| Compartment | Period | PC (copies/mL) | | | | | | | | | | | | | | |
| Lumen | Control 1 | 1,19E+10 | ± | 8,69E+08 | 5,18E+07 | ± | 3,13E+06 | 6,05E+09 | ± | 2,67E+08 | 5,28E+05 | ± | 4,81E+04 | 1,77E+10 | ± | 1,04E+09 |
|  | Control 2 | 6,93E+09 | ± | 5,84E+08 | 1,09E+08 | ± | 8,75E+05 | 4,70E+09 | ± | 1,71E+08 | 4,81E+05 | ± | 1,60E+04 | 1,74E+10 | ± | 1,49E+09 |
|  | Treatment 1 | 6,49E+09 | ± | 7,66E+08 | 3,76E+07 | ± | 3,50E+06 | 5,98E+09 | ± | 9,04E+08 | 4,43E+05 | ± | 9,81E+04 | 2,58E+10 | ± | 4,38E+08 |
|  | Treatment 2 | 6,49E+09 | ± | 8,03E+08 | 3,76E+07 | ± | 3,48E+06 | 5,98E+09 | ± | 9,11E+08 | 4,43E+05 | ± | 4,92E+04 | 2,58E+10 | ± | 2,13E+09 |
|  | Treatment 3 | 9,59E+09 | ± | 1,78E+08 | 2,46E+08 | ± | 1,99E+07 | 4,41E+09 | ± | 4,74E+08 | 4,87E+05 | ± | 2,55E+04 | 9,26E+09 | ± | 1,95E+08 |
|  | Wash Out 1 | 6,00E+09 | ± | 6,40E+08 | 2,84E+07 | ± | 1,38E+06 | 4,74E+09 | ± | 4,53E+08 | 6,07E+05 | ± | 5,48E+04 | 2,10E+10 | ± | 6,27E+08 |
|  | Wash Out 2 | 5,40E+09 | ± | 7,73E+08 | 3,79E+07 | ± | 7,23E+06 | 4,31E+09 | ± | 5,73E+08 | 4,59E+05 | ± | 4,33E+04 | 1,87E+10 | ± | 2,76E+09 |
| Mucus | Control 1 | 4.99E+08 | ± | 4.83E+07 | 9.57E+06 | ± | 9.15E+05 | 9.08E+09 | ± | 2.23E+08 | 0.00E+00 | ± | 0.00E+00 | 2.01E+09 | ± | 3.94E+07 |
|  | Control 2 | 1.03E+09 | ± | 3.85E+07 | 2.92E+07 | ± | 1.03E+06 | 2.06E+10 | ± | 4.20E+08 | 0.00E+00 | ± | 0.00E+00 | 3.76E+09 | ± | 6.30E+08 |
|  | Treatment 1 | 1.85E+09 | ± | 9.45E+07 | 3.68E+07 | ± | 4.90E+06 | 5.46E+10 | ± | 2.72E+09 | 0.00E+00 | ± | 0.00E+00 | 4.15E+09 | ± | 6.11E+08 |
|  | Treatment 2 | 1.85E+09 | ± | 1.11E+08 | 3.68E+07 | ± | 2.94E+06 | 5.46E+10 | ± | 3.29E+09 | 0.00E+00 | ± | 0.00E+00 | 4.15E+09 | ± | 1.68E+08 |
|  | Treatment 3 | 5.97E+09 | ± | 1.88E+08 | 1.74E+08 | ± | 2.60E+07 | 2.00E+11 | ± | 4.33E+09 | 0.00E+00 | ± | 0.00E+00 | 5.58E+09 | ± | 1.59E+08 |
|  | Wash Out 1 | 1.79E+09 | ± | 6.94E+07 | 2.48E+07 | ± | 1.89E+06 | 4.30E+10 | ± | 5.66E+09 | 5.93E+03 | ± | 2.81E+03 | 6.19E+09 | ± | 7.10E+08 |
|  | Wash Out 2 | 2.19E+09 | ± | 1.15E+08 | 1.02E+08 | ± | 2.60E+06 | 4.03E+10 | ± | 2.20E+09 | 0.00E+00 | ± | 0.00E+00 | 7.86E+09 | ± | 8.04E+08 |
|  |  | DC (copies/mL) | | | | | | | | | | | | | | |
| Lumen | Control 1 | 1,74E+10 | ± | 1,91E+09 | 8,70E+09 | ± | 7,17E+08 | 7,42E+09 | ± | 1,67E+09 | 2,72E+05 | ± | 3,36E+04 | 1,92E+10 | ± | 5,66E+08 |
|  | Control 2 | 1,63E+10 | ± | 8,41E+07 | 8,28E+09 | ± | 4,69E+08 | 6,82E+09 | ± | 4,81E+08 | 3,18E+05 | ± | 5,88E+04 | 1,33E+10 | ± | 9,93E+07 |
|  | Treatment 1 | 1,93E+10 | ± | 1,21E+09 | 6,81E+09 | ± | 1,59E+08 | 1,28E+10 | ± | 7,26E+08 | 2,27E+05 | ± | 1,15E+04 | 2,03E+10 | ± | 6,49E+08 |
|  | Treatment 2 | 2,16E+10 | ± | 2,13E+09 | 6,41E+09 | ± | 5,39E+08 | 1,27E+10 | ± | 5,36E+08 | 1,92E+05 | ± | 1,75E+04 | 6,44E+09 | ± | 2,49E+08 |
|  | Treatment 3 | 2,53E+10 | ± | 5,85E+08 | 1,01E+10 | ± | 3,64E+08 | 9,41E+09 | ± | 3,97E+08 | 8,57E+04 | ± | 2,91E+03 | 2,53E+09 | ± | 5,43E+07 |
|  | Wash Out 1 | 2,01E+10 | ± | 2,91E+09 | 6,99E+09 | ± | 3,16E+08 | 8,03E+09 | ± | 5,62E+08 | 9,79E+04 | ± | 4,16E+04 | 5,78E+09 | ± | 7,42E+08 |
|  | Wash Out 2 | 2,19E+10 | ± | 2,70E+09 | 8,72E+09 | ± | 2,20E+08 | 9,14E+09 | ± | 6,50E+08 | 1,45E+05 | ± | 1,84E+04 | 1,25E+10 | ± | 2,73E+08 |
| Mucus | Control 1 | 1.13E+09 | ± | 1.98E+08 | 3.06E+08 | ± | 7.22E+07 | 2.05E+10 | ± | 8.07E+08 | 0.00E+00 | ± | 0.00E+00 | 1.05E+09 | ± | 1.58E+08 |
|  | Control 2 | 1.64E+09 | ± | 2.14E+07 | 4.29E+08 | ± | 1.34E+07 | 3.06E+10 | ± | 3.35E+08 | 0.00E+00 | ± | 0.00E+00 | 1.24E+09 | ± | 1.09E+07 |
|  | Treatment 1 | 3.65E+09 | ± | 2.02E+07 | 4.40E+08 | ± | 3.66E+07 | 9.09E+10 | ± | 1.88E+09 | 1.31E+05 | ± | 4.87E+04 | 2.74E+09 | ± | 4.89E+07 |
|  | Treatment 2 | 3.16E+09 | ± | 1.26E+08 | 6.83E+08 | ± | 7.32E+07 | 6.64E+10 | ± | 6.70E+08 | 4.99E+04 | ± | 1.26E+04 | 9.12E+08 | ± | 1.96E+07 |
|  | Treatment 3 | 6.32E+09 | ± | 5.71E+07 | 7.36E+08 | ± | 5.19E+07 | 2.03E+11 | ± | 2.06E+09 | 5.28E+03 | ± | 2.60E+03 | 3.16E+08 | ± | 1.82E+07 |
|  | Wash Out 1 | 2.29E+09 | ± | 1.24E+08 | 9.14E+08 | ± | 2.84E+07 | 3.43E+10 | ± | 1.66E+09 | 5.98E+04 | ± | 2.37E+04 | 2.25E+08 | ± | 5.41E+06 |
|  | Wash Out 2 | 2.06E+09 | ± | 6.02E+07 | 8.89E+08 | ± | 2.81E+07 | 2.96E+10 | ± | 9.44E+08 | 0.00E+00 | ± | 0.00E+00 | 9.53E+08 | ± | 6.11E+07 |

Table S11. Luminal microbial community composition in the proximal colon (PC) of the baby M-SHIME**^®^** experiment (donor baby B) during the control, treatment and washout periods (1.5, 3 and 4.5 g/d SCF) as assessed by qPCR. All samples were analyzed in technical triplicates. Data are shown as mean ± standard deviation. PC = proximal colon. SCF = soluble corn fiber. M-SHIME = mucosal simulator of the human intestinal microbial ecosystem

|  |  | Total bacteria | | | Bacteroidetes group | | | Firmicutes group | | | Lactobacilli | | | Bifidobacteria | | |
| --- | --- | --- | --- | --- | --- | --- | --- | --- | --- | --- | --- | --- | --- | --- | --- | --- |
| Treatment (SCF) | Period | PC - LUMEN (copies/mL) | | | | | | | | | | | | | | |
| 1.5 g/d | Control 1 | 1.55E+10 | ± | 1.02E+09 | 1.09E+06 | ± | 1.50E+05 | 2.53E+09 | ± | 4.03E+08 | 7.09E+04 | ± | 8.63E+04 | 3.26E+09 | ± | 1.95E+08 |
|  | Control 2 | 1.67E+10 | ± | 1.90E+09 | 1.62E+05 | ± | 7.83E+03 | 2.74E+09 | ± | 8.76E+07 | 2.68E+05 | ± | 1.12E+05 | 5.11E+09 | ± | 2.66E+08 |
|  | Treatment 1 | 2.54E+10 | ± | 7.43E+08 | 3.10E+05 | ± | 3.58E+04 | 3.58E+09 | ± | 1.28E+08 | 1.92E+05 | ± | 1.63E+05 | 7.00E+09 | ± | 3.32E+08 |
|  | Treatment 2 | 2.20E+10 | ± | 4.73E+08 | 1.69E+05 | ± | 4.35E+04 | 4.28E+09 | ± | 2.25E+08 | 4.44E+04 | ± | 6.16E+04 | 1.53E+10 | ± | 1.49E+09 |
|  | Treatment 3 | 2.19E+10 | ± | 5.08E+08 | 1.34E+05 | ± | 0.00E+00 | 3.79E+09 | ± | 9.60E+08 | 2.00E+05 | ± | 1.54E+05 | 1.17E+10 | ± | 1.94E+08 |
|  | Wash Out 1 | 1.36E+10 | ± | 1.45E+08 | 1.01E+06 | ± | 1.38E+05 | 2.46E+09 | ± | 9.90E+07 | 3.06E+05 | ± | 1.16E+05 | 6.33E+09 | ± | 6.66E+07 |
|  | Wash Out 2 | 1.84E+10 | ± | 2.10E+08 | 2.08E+05 | ± | 1.79E+04 | 3.32E+09 | ± | 3.13E+08 | 5.43E+04 | ± | 6.53E+03 | 7.32E+09 | ± | 1.17E+08 |
| 3 g/d | Control 1 | 6.44E+09 | ± | 9.34E+08 | 1.76E+08 | ± | 1.75E+07 | 7.90E+09 | ± | 4.19E+08 | 1.28E+05 | ± | 2.29E+04 | 1.98E+09 | ± | 2.34E+08 |
|  | Control 2 | 2.14E+09 | ± | 9.40E+07 | 8.75E+07 | ± | 4.34E+06 | 4.02E+09 | ± | 9.58E+07 | 3.87E+04 | ± | 4.49E+03 | 1.00E+09 | ± | 4.51E+07 |
|  | Treatment 1 | 1.21E+10 | ± | 3.18E+08 | 4.92E+08 | ± | 2.05E+07 | 2.25E+10 | ± | 7.92E+08 | 2.31E+05 | ± | 1.48E+04 | 3.13E+09 | ± | 7.06E+07 |
|  | Treatment 2 | 1.50E+10 | ± | 6.19E+07 | 8.24E+08 | ± | 1.19E+07 | 2.60E+10 | ± | 2.37E+09 | 3.11E+05 | ± | 3.97E+04 | 4.90E+09 | ± | 8.81E+07 |
|  | Treatment 3 | 7.15E+09 | ± | 8.79E+08 | 2.60E+08 | ± | 1.14E+07 | 1.33E+10 | ± | 3.81E+08 | 1.68E+05 | ± | 2.19E+04 | 3.26E+09 | ± | 2.07E+08 |
|  | Wash Out 1 | 7.39E+09 | ± | 2.04E+08 | 4.46E+08 | ± | 1.45E+07 | 1.41E+10 | ± | 1.28E+09 | 4.66E+05 | ± | 6.56E+04 | 2.79E+09 | ± | 1.49E+08 |
|  | Wash Out 2 | 9.54E+09 | ± | 1.81E+08 | 4.88E+08 | ± | 2.29E+07 | 1.75E+10 | ± | 2.95E+08 | 1.77E+05 | ± | 4.77E+04 | 4.25E+09 | ± | 1.37E+08 |
| 4.5 g/d | Control 1 | 6.42E+09 | ± | 7.17E+08 | 5.07E+06 | ± | 5.97E+05 | 8.02E+09 | ± | 6.78E+08 | 2.12E+04 | ± | 6.14E+03 | 2.38E+09 | ± | 1.53E+08 |
|  | Control 2 | 1.24E+10 | ± | 2.16E+08 | 1.28E+08 | ± | 2.75E+06 | 1.78E+10 | ± | 6.85E+08 | 3.36E+04 | ± | 6.27E+03 | 4.09E+09 | ± | 1.84E+08 |
|  | Treatment 1 | 1.41E+10 | ± | 4.86E+08 | 1.01E+07 | ± | 1.96E+05 | 1.80E+10 | ± | 4.78E+08 | 9.59E+04 | ± | 3.06E+04 | 3.60E+09 | ± | 8.57E+07 |
|  | Treatment 2 | 1.04E+10 | ± | 3.96E+08 | 2.02E+06 | ± | 1.00E+05 | 1.58E+10 | ± | 1.59E+09 | 8.56E+05 | ± | 1.51E+05 | 4.88E+09 | ± | 1.83E+08 |
|  | Treatment 3 | 1.08E+10 | ± | 1.94E+08 | 1.29E+05 | ± | 2.82E+03 | 2.17E+10 | ± | 1.24E+09 | 1.45E+07 | ± | 4.73E+05 | 6.14E+09 | ± | 9.38E+07 |
|  | Wash Out 1 | 1.43E+10 | ± | 2.69E+08 | 3.23E+07 | ± | 7.21E+05 | 2.01E+10 | ± | 1.18E+09 | 1.54E+07 | ± | 4.59E+05 | 5.61E+09 | ± | 1.32E+08 |
|  | Wash Out 2 | 8.83E+09 | ± | 5.28E+08 | 1.54E+08 | ± | 7.83E+06 | 1.68E+10 | ± | 8.48E+08 | 2.07E+07 | ± | 7.00E+05 | 3.36E+09 | ± | 2.30E+07 |

Table S12. Luminal microbial community composition in the distal colon (DC) of the baby M-SHIME**^®^** experiment (donor baby B) during the control, treatment and washout periods (1.5, 3 and 4.5 g/d SCF) as assessed by qPCR. All samples were analyzed in technical triplicates. Data are shown as mean ± standard deviation. DC = distal colon. SCF = soluble corn fiber. M-SHIME = mucosal simulator of the human intestinal microbial ecosystem

|  |  | Total bacteria | | | Bacteroidetes group | | | Firmicutes group | | | Lactobacilli | | | Bifidobacteria | | |
| --- | --- | --- | --- | --- | --- | --- | --- | --- | --- | --- | --- | --- | --- | --- | --- | --- |
| Treatment (SCF) | Period | DC - LUMEN (copies/mL) | | | | | | | | | | | | | | |
| 1.5 g/d | Control 1 | 2.74E+10 | ± | 3.38E+09 | 4.06E+09 | ± | 7.57E+08 | 5.68E+09 | ± | 4.36E+08 | 6.08E+04 | ± | 4.11E+04 | 4.09E+09 | ± | 2.38E+08 |
|  | Control 2 | 4.19E+10 | ± | 4.34E+08 | 8.51E+09 | ± | 1.28E+08 | 9.79E+09 | ± | 2.58E+08 | 2.43E+04 | ± | 2.06E+04 | 5.33E+09 | ± | 6.91E+07 |
|  | Treatment 1 | 1.60E+10 | ± | 1.27E+08 | 3.87E+09 | ± | 1.60E+08 | 3.84E+09 | ± | 1.21E+08 | 4.08E+04 | ± | 3.38E+04 | 2.42E+09 | ± | 1.66E+08 |
|  | Treatment 2 | 1.35E+10 | ± | 2.63E+08 | 3.50E+09 | ± | 5.36E+07 | 3.36E+09 | ± | 1.79E+08 | 4.49E+04 | ± | 7.14E+03 | 2.94E+09 | ± | 3.34E+07 |
|  | Treatment 3 | 2.47E+10 | ± | 2.59E+08 | 7.51E+09 | ± | 1.29E+08 | 6.69E+09 | ± | 2.02E+08 | 5.61E+04 | ± | 2.45E+04 | 2.75E+09 | ± | 1.87E+08 |
|  | Wash Out 1 | 2.45E+10 | ± | 4.93E+08 | 5.49E+09 | ± | 9.39E+07 | 5.90E+09 | ± | 6.26E+08 | 1.12E+05 | ± | 1.05E+05 | 2.85E+09 | ± | 8.19E+07 |
|  | Wash Out 2 | 3.83E+10 | ± | 2.39E+09 | 7.75E+09 | ± | 2.52E+08 | 8.33E+09 | ± | 8.77E+07 | 5.53E+04 | ± | 4.99E+04 | 5.53E+09 | ± | 1.92E+08 |
| 3 g/d | Control 1 | 1.37E+10 | ± | 1.08E+09 | 1.22E+09 | ± | 8.57E+07 | 2.79E+10 | ± | 2.50E+09 | 8.06E+04 | ± | 2.64E+04 | 1.98E+09 | ± | 2.56E+08 |
|  | Control 2 | 1.02E+10 | ± | 8.57E+08 | 1.55E+09 | ± | 8.78E+07 | 2.28E+10 | ± | 8.99E+08 | 8.07E+04 | ± | 1.40E+04 | 1.41E+09 | ± | 4.18E+07 |
|  | Treatment 1 | 1.56E+10 | ± | 5.72E+08 | 2.29E+09 | ± | 7.47E+07 | 4.00E+10 | ± | 1.31E+09 | 6.24E+04 | ± | 1.14E+04 | 1.47E+09 | ± | 3.01E+07 |
|  | Treatment 2 | 1.29E+10 | ± | 3.48E+08 | 1.79E+09 | ± | 5.54E+07 | 3.20E+10 | ± | 1.46E+09 | 4.94E+04 | ± | 1.62E+04 | 1.47E+09 | ± | 6.14E+07 |
|  | Treatment 3 | 8.28E+09 | ± | 2.22E+08 | 1.03E+09 | ± | 6.45E+07 | 2.24E+10 | ± | 1.43E+09 | 2.24E+04 | ± | 2.81E+03 | 1.20E+09 | ± | 2.95E+07 |
|  | Wash Out 1 | 3.90E+09 | ± | 1.11E+08 | 4.36E+08 | ± | 8.18E+06 | 1.07E+10 | ± | 3.97E+08 | 8.92E+04 | ± | 2.11E+04 | 9.33E+08 | ± | 7.13E+07 |
|  | Wash Out 2 | 7.39E+09 | ± | 1.62E+08 | 8.61E+08 | ± | 8.30E+06 | 1.73E+10 | ± | 3.77E+08 | 5.99E+04 | ± | 1.31E+04 | 1.65E+09 | ± | 6.51E+07 |
| 4.5 g/d | Control 1 | 1.09E+10 | ± | 7.25E+08 | 1.25E+09 | ± | 1.00E+08 | 2.71E+10 | ± | 2.57E+09 | 1.01E+04 | ± | 3.58E+03 | 2.07E+09 | ± | 4.20E+08 |
|  | Control 2 | 1.59E+10 | ± | 1.16E+09 | 2.28E+09 | ± | 1.26E+08 | 3.88E+10 | ± | 1.55E+08 | 1.07E+04 | ± | 8.18E+03 | 2.37E+09 | ± | 1.23E+08 |
|  | Treatment 1 | 1.81E+10 | ± | 7.45E+08 | 1.75E+09 | ± | 1.67E+07 | 5.23E+10 | ± | 3.72E+09 | 4.89E+04 | ± | 1.40E+04 | 2.49E+09 | ± | 1.10E+08 |
|  | Treatment 2 | 2.48E+10 | ± | 4.06E+08 | 1.97E+09 | ± | 3.19E+07 | 7.46E+10 | ± | 5.43E+09 | 6.88E+05 | ± | 6.73E+04 | 5.24E+09 | ± | 1.87E+08 |
|  | Treatment 3 | 2.02E+10 | ± | 4.54E+08 | 1.79E+09 | ± | 1.85E+08 | 6.40E+10 | ± | 4.72E+09 | 2.30E+06 | ± | 2.82E+05 | 4.50E+09 | ± | 1.84E+08 |
|  | Wash Out 1 | 2.13E+10 | ± | 3.63E+08 | 2.67E+09 | ± | 1.20E+08 | 6.69E+10 | ± | 7.98E+09 | 7.06E+06 | ± | 1.97E+04 | 3.15E+09 | ± | 7.32E+07 |
|  | Wash Out 2 | 2.32E+10 | ± | 4.57E+08 | 2.24E+09 | ± | 9.72E+07 | 6.61E+10 | ± | 2.28E+09 | 8.51E+06 | ± | 1.17E+05 | 3.15E+09 | ± | 1.56E+08 |

Table S13. Mucosal microbial community composition in the proximal colon of the baby M-SHIME**^®^** experiment (donor baby B) during the control, treatment and washout periods (1.5, 3 and 4.5 g/d SCF) as assessed by qPCR. All samples were analyzed in technical triplicates. Data are shown as mean ± standard deviation. PC = proximal colon. SCF = soluble corn fiber. M-SHIME = mucosal simulator of the human intestinal microbial ecosystem

|  |  | Total bacteria | | | Bacteroidetes group | | | Firmicutes group | | | | Lactobacilli | | | | Bifidobacteria | | | |
| --- | --- | --- | --- | --- | --- | --- | --- | --- | --- | --- | --- | --- | --- | --- | --- | --- | --- | --- | --- |
| Treatment (SCF) | Period | PC -MUCUS (copies/mL) | | | | | | | | | | | | | | | | | |
| 1.5 g/d | Control 1 | 4.82E+09 | ± | 4.03E+07 | 1.59E+06 | ± | 2.18E+05 | 2.84E+09 | ± | 4.65E+07 | 2.24E+02 | | ± | 0.00E+00 | 5.89E+09 | | ± | 5.71E+08 |  |
|  | Control 2 | 2.71E+08 | ± | 5.91E+07 | 5.17E+05 | ± | 8.70E+04 | 2.34E+08 | ± | 2.88E+07 | 3.58E+03 | | ± | 1.70E+03 | 3.26E+08 | | ± | 2.95E+07 |  |
|  | Treatment 1 | 3.11E+09 | ± | 3.11E+08 | 5.07E+06 | ± | 5.59E+05 | 1.49E+09 | ± | 8.45E+07 | 6.48E+03 | | ± | 2.96E+03 | 3.94E+09 | | ± | 1.11E+08 |  |
|  | Treatment 2 | 4.29E+09 | ± | 2.21E+08 | 3.42E+06 | ± | 2.00E+05 | 2.19E+09 | ± | 1.38E+08 | 1.11E+03 | | ± | 7.70E+02 | 1.04E+10 | | ± | 7.14E+08 |  |
|  | Treatment 3 | 2.74E+09 | ± | 9.17E+07 | 1.37E+06 | ± | 9.72E+04 | 1.57E+09 | ± | 3.45E+07 | 2.21E+04 | | ± | 3.98E+03 | 8.04E+09 | | ± | 1.09E+08 |  |
|  | Wash Out 1 | 3.53E+09 | ± | 4.65E+07 | 7.81E+06 | ± | 2.19E+05 | 3.05E+09 | ± | 1.22E+08 | 6.11E+02 | | ± | 1.94E+02 | 5.21E+09 | | ± | 3.36E+08 |  |
|  | Wash Out 2 | 2.44E+09 | ± | 3.38E+07 | 2.98E+06 | ± | 2.28E+05 | 1.84E+09 | ± | 4.40E+08 | 3.33E+04 | | ± | 1.11E+04 | 3.90E+09 | | ± | 1.36E+08 |  |
| 3 g/d | Control 1 | 8.69E+07 | ± | 1.15E+07 | 1.94E+07 | ± | 1.23E+06 | 3.47E+08 | ± | 5.33E+07 | 9.61E+02 | | ± | 2.38E+02 | 2.49E+07 | | ± | 2.80E+06 |  |
|  | Control 2 | 1.95E+08 | ± | 2.48E+07 | 5.34E+07 | ± | 4.41E+06 | 8.97E+08 | ± | 7.50E+07 | 2.52E+03 | | ± | 1.87E+03 | 3.98E+07 | | ± | 3.68E+05 |  |
|  | Treatment 1 | 3.46E+09 | ± | 4.57E+07 | 5.21E+08 | ± | 2.56E+07 | 2.28E+10 | ± | 7.26E+08 | 3.10E+03 | | ± | 2.72E+03 | 6.40E+08 | | ± | 1.21E+07 |  |
|  | Treatment 2 | 1.62E+09 | ± | 2.15E+07 | 3.18E+08 | ± | 3.82E+06 | 1.26E+10 | ± | 2.93E+08 | 3.27E+03 | | ± | 2.40E+03 | 2.63E+08 | | ± | 5.54E+06 |  |
|  | Treatment 3 | 1.76E+09 | ± | 1.37E+08 | 2.84E+08 | ± | 1.04E+07 | 1.31E+10 | ± | 6.60E+08 | 5.39E+04 | | ± | 1.07E+04 | 3.71E+08 | | ± | 1.69E+07 |  |
|  | Wash Out 1 | 1.05E+09 | ± | 1.38E+07 | 2.53E+08 | ± | 6.84E+06 | 8.32E+09 | ± | 1.79E+08 | 6.89E+04 | | ± | 1.28E+04 | 1.24E+08 | | ± | 7.04E+06 |  |
|  | Wash Out 2 | 7.12E+08 | ± | 3.16E+07 | 1.80E+08 | ± | 8.96E+06 | 5.57E+09 | ± | 1.37E+09 | 6.62E+04 | | ± | 1.85E+04 | 9.24E+07 | | ± | 1.47E+07 |  |
| 4.5 g/d | Control 1 | 9.51E+09 | ± | 1.01E+09 | 2.21E+07 | ± | 2.87E+05 | 3.40E+09 | ± | 1.08E+08 | 1.58E+02 | | ± | 0.00E+00 | 9.77E+09 | | ± | 9.28E+08 |  |
|  | Control 2 | 1.59E+10 | ± | 4.17E+08 | 3.10E+08 | ± | 5.40E+06 | 4.97E+09 | ± | 1.21E+08 | 7.65E+02 | | ± | 2.77E+02 | 1.68E+10 | | ± | 3.20E+08 |  |
|  | Treatment 1 | 3.01E+10 | ± | 5.87E+08 | 2.97E+08 | ± | 1.13E+07 | 7.15E+09 | ± | 3.97E+08 | <LOQ | | | | 2.50E+10 | | ± | 5.26E+08 |  |
|  | Treatment 2 | 1.80E+10 | ± | 1.90E+09 | 8.48E+06 | ± | 2.27E+05 | 6.80E+09 | ± | 1.23E+08 | 9.39E+02 | | ± | 7.27E+02 | 2.54E+10 | | ± | 1.96E+08 |  |
|  | Treatment 3 | 1.48E+10 | ± | 5.27E+08 | 4.21E+06 | ± | 1.34E+05 | 6.49E+09 | ± | 1.40E+08 | 3.37E+03 | | ± | 2.43E+03 | 1.25E+10 | | ± | 5.79E+08 |  |
|  | Wash Out 1 | 1.51E+10 | ± | 3.02E+08 | 1.00E+08 | ± | 8.14E+06 | 7.69E+09 | ± | 2.38E+08 | 3.73E+02 | | ± | 1.92E+02 | 6.67E+09 | | ± | 7.75E+08 |  |
|  | Wash Out 2 | 7.80E+09 | ± | 1.45E+08 | 2.31E+08 | ± | 6.42E+06 | 3.41E+09 | ± | 2.54E+07 | 9.69E+04 | | ± | 6.45E+04 | 3.34E+09 | | ± | 1.70E+08 |  |

Table S14. Mucosal microbial community composition in the distal colon of the baby M-SHIME**^®^** experiment (donor baby B) during the control, treatment and washout periods (1.5, 3 and 4.5 g/d SCF) as assessed by qPCR. Data represent mean ± standard deviation of the mean (n=3 time points per period and n=3 technical replicates per sample). DC = distal colon. SCF = soluble corn fiber. M-SHIME = mucosal simulator of the human intestinal microbial ecosystem

|  |  | Total bacteria | | | Bacteroidetes group | | | Firmicutes group | | | | Lactobacilli | | | | Bifidobacteria | | |
| --- | --- | --- | --- | --- | --- | --- | --- | --- | --- | --- | --- | --- | --- | --- | --- | --- | --- | --- |
| Treatment (SCF) | Period | DC -MUCUS (copies/mL) | | | | | | | | | | | | | | | | |
| 1.5 g/d | Control 1 | 3.67E+08 | ± | 3.93E+07 | 7.49E+07 | ± | 3.79E+06 | 5.84E+08 | ± | 6.91E+07 | 2.32E+03 | | ± | 1.57E+03 | 2.53E+07 | | ± | 3.71E+06 |
|  | Control 2 | 3.71E+09 | ± | 1.45E+08 | 1.68E+09 | ± | 8.86E+07 | 4.60E+09 | ± | 5.51E+08 | <LOQ | | | | 4.93E+08 | | ± | 1.57E+07 |
|  | Treatment 1 | 3.67E+08 | ± | 1.24E+07 | 1.36E+08 | ± | 2.19E+06 | 4.68E+08 | ± | 2.41E+07 | 3.35E+03 | | ± | 3.43E+03 | 3.76E+07 | | ± | 2.26E+06 |
|  | Treatment 2 | 6.14E+09 | ± | 1.21E+08 | 3.37E+09 | ± | 1.86E+08 | 7.03E+09 | ± | 3.85E+08 | 1.65E+03 | | ± | 4.95E+02 | 1.31E+09 | | ± | 5.34E+07 |
|  | Treatment 3 | 4.58E+09 | ± | 1.63E+07 | 3.04E+09 | ± | 1.12E+08 | 4.94E+09 | ± | 2.82E+08 | 1.52E+04 | | ± | 9.54E+03 | 7.41E+08 | | ± | 1.01E+08 |
|  | Wash Out 1 | 4.48E+09 | ± | 2.15E+08 | 2.50E+09 | ± | 1.37E+08 | 4.95E+09 | ± | 1.32E+09 | 1.62E+03 | | ± | 7.25E+02 | 6.74E+08 | | ± | 1.02E+07 |
|  | Wash Out 2 | 1.65E+08 | ± | 2.62E+06 | 9.25E+07 | ± | 7.02E+06 | 2.00E+08 | ± | 5.43E+06 | 2.17E+04 | | ± | 3.88E+03 | 5.98E+07 | | ± | 3.31E+06 |
| 3 g/d | Control 1 | 1.07E+08 | ± | 3.37E+06 | 3.34E+07 | ± | 1.48E+06 | 7.57E+08 | ± | 6.10E+07 | 7.95E+02 | | ± | 3.21E+02 | 5.36E+06 | | ± | 3.42E+05 |
|  | Control 2 | 4.53E+09 | ± | 1.36E+08 | 1.34E+09 | ± | 8.41E+07 | 3.77E+10 | ± | 7.29E+09 | 3.43E+02 | | ± | 0.00E+00 | 2.07E+08 | | ± | 5.81E+06 |
|  | Treatment 1 | 7.96E+09 | ± | 2.95E+08 | 2.02E+09 | ± | 8.37E+07 | 5.62E+10 | ± | 4.08E+09 | 2.38E+03 | | ± | 1.76E+03 | 7.00E+08 | | ± | 4.14E+07 |
|  | Treatment 2 | 9.71E+08 | ± | 3.16E+07 | 4.11E+08 | ± | 2.49E+07 | 7.35E+09 | ± | 7.21E+08 | 1.06E+03 | | ± | 7.19E+02 | 4.80E+07 | | ± | 8.49E+05 |
|  | Treatment 3 | 6.34E+08 | ± | 9.95E+07 | 3.23E+08 | ± | 5.16E+06 | 4.51E+09 | ± | 2.67E+08 | 2.42E+04 | | ± | 6.35E+03 | 5.12E+07 | | ± | 5.01E+06 |
|  | Wash Out 1 | 5.18E+08 | ± | 2.02E+07 | 1.79E+08 | ± | 3.67E+06 | 3.91E+09 | ± | 3.20E+08 | 5.53E+04 | | ± | 1.63E+04 | 4.03E+07 | | ± | 4.57E+06 |
|  | Wash Out 2 | 2.70E+07 | ± | 7.52E+05 | 1.71E+07 | ± | 5.96E+05 | 2.28E+08 | ± | 1.80E+07 | 1.10E+04 | | ± | 2.21E+03 | 1.47E+06 | | ± | 9.66E+04 |
| 4.5 g/d | Control 1 | 1.25E+10 | ± | 4.14E+08 | 6.68E+08 | ± | 2.06E+07 | 9.22E+09 | ± | 1.24E+09 | <LOQ | | | | 1.53E+09 | | ± | 2.47E+08 |
|  | Control 2 | 1.16E+10 | ± | 6.48E+08 | 8.92E+08 | ± | 1.29E+08 | 7.53E+09 | ± | 5.07E+08 | <LOQ | | | | 2.39E+09 | | ± | 1.44E+08 |
|  | Treatment 1 | 8.91E+08 | ± | 1.56E+07 | 6.24E+07 | ± | 1.12E+06 | 6.47E+08 | ± | 2.04E+07 | <LOQ | | | | 6.97E+07 | | ± | 2.93E+06 |
|  | Treatment 2 | 2.99E+10 | ± | 2.61E+09 | 2.32E+09 | ± | 1.96E+08 | 1.52E+10 | ± | 4.71E+08 | 2.78E+03 | | ± | 7.86E+02 | 6.28E+09 | | ± | 6.69E+08 |
|  | Treatment 3 | 1.35E+09 | ± | 4.05E+07 | 8.54E+07 | ± | 4.12E+06 | 9.47E+08 | ± | 5.35E+07 | 1.14E+04 | | ± | 1.77E+03 | 1.41E+08 | | ± | 8.05E+06 |
|  | Wash Out 1 | 9.34E+09 | ± | 1.71E+08 | 7.48E+08 | ± | 8.85E+06 | 5.78E+09 | ± | 3.01E+08 | 3.09E+04 | | ± | 5.77E+03 | 1.08E+09 | | ± | 5.72E+07 |
|  | Wash Out 2 | 3.39E+08 | ± | 6.85E+06 | 3.39E+07 | ± | 5.06E+05 | 2.25E+08 | ± | 2.77E+06 | 1.09E+04 | | ± | 3.23E+03 | 1.58E+07 | | ± | 1.29E+06 |

Table S15. Statistical analysis (3-way ANOVA) of SCF effect on major microbial taxa of infant gut microbiota in baby C and baby D, during the control period (C) or different weeks of SCF treatment (week 1 = W1; week 2 = W2; week 3 = W3), in the mucus and luminal compartment.

|  |  |  | Lumen | | | Mucus | | |
| --- | --- | --- | --- | --- | --- | --- | --- | --- |
| Tukey's multiple comparisons test | | | Bacteroidetes | Firmicutes | Bifidobacetria | Bacteroidetes | Firmicutes | Bifidobacetria |
|  |  |  | Summary | Summary | Summary | Summary | Summary | Summary |
|  | PC | Baby C vs. Baby D | **** | **** | *** | ns | *** | ** |
|  | DC | Baby C vs. Baby D | **** | **** | **** | ns | ** | * |
| Baby C | PC | Control vs. SCF_W1 | **** | **** | **** | ns | ns | ns |
|  |  | Control vs. SCF_W2 | ns | ** | ns | **** | **** | **** |
|  |  | Control vs. SCF_W3 | ns | ** | ns | **** | ** | **** |
|  |  | SCF_W1 vs. SCF_W2 | **** | **** | **** | **** | **** | **** |
|  |  | SCF_W2 vs. SCF_W3 | **** | **** | **** | **** | ** | **** |
|  |  | SCF_W1 vs. SCF_W3 | ns | ns | ns | ** | ns | ns |
|  | DC | Control vs. SCF_W1 | ns | ns | ns | **** | ** | ns |
|  |  | Control vs. SCF_W2 | ns | ns | **** | * | * | ** |
|  |  | Control vs. SCF_W3 | ns | * | ** | ns | ns | ns |
|  |  | SCF_W1 vs. SCF_W2 | ** | ns | **** | **** | ns | ns |
|  |  | SCF_W2 vs. SCF_W3 | * | ns | *** | **** | ns | ns |
|  |  | SCF_W1 vs. SCF_W3 | ns | ns | **** | ns | ns | ns |
| Baby D | PC | Control vs. SCF_W1 | ns | ns | ns | **** | **** | ns |
|  |  | Control vs. SCF_W2 | ns | ns | ns | **** | * | ns |
|  |  | Control vs. SCF_W3 | ns | ns | ns | **** | **** | **** |
|  |  | SCF_W1 vs. SCF_W2 | ns | ns | ns | **** | ns | ns |
|  |  | SCF_W2 vs. SCF_W3 | ns | ns | ns | **** | ns | ** |
|  |  | SCF_W1 vs. SCF_W3 | ns | ns | ns | **** | * | * |
|  | DC | Control vs. SCF_W1 | *** | ns | ns | ** | ns | ns |
|  |  | Control vs. SCF_W2 | ns | ns | ns | ns | ns | ns |
|  |  | Control vs. SCF_W3 | ns | ns | ns | **** | ns | ns |
|  |  | SCF_W1 vs. SCF_W2 | ns | ns | ns | ns | ns | ns |
|  |  | SCF_W2 vs. SCF_W3 | ns | ns | ns | ns | ns | ns |
|  |  | SCF_W1 vs. SCF_W3 | ns | ns | ns | **** | ns | ns |

Table S16. Short chain fatty acid (SCFA) production during the baby M-SHIME experiment (donor A, 1.5g/d SCF). Data represent geometric mean ± standard deviation (n=3 time points per week) of SCFA at different time points of the assay.

|  |  | Short chain fatty acids (mmol/L) | | | | | | | | | | | |
| --- | --- | --- | --- | --- | --- | --- | --- | --- | --- | --- | --- | --- | --- |
|  |  | Ascending | | | | | | | | | | | |
|  | **Week (W)** | Acetate | | | Propionate | | | Butyrate | | | Total SCFA | | |
| AC | **Control 1 (W1)** | 8,73 | ± | 2,11 | 4,04 | ± | 0,48 | 4,87 | ± | 0,55 | 19,37 | ± | 2,73 |
|  | **Control 2 (W2)** | 9,05 | ± | 2,56 | 4,17 | ± | 0,30 | 5,87 | ± | 0,87 | 21,39 | ± | 3,92 |
|  | **Treatment 1 (W3)** | 10,00 | ± | 0,67 | 4,24 | ± | 0,39 | 6,92 | ± | 0,47 | 23,53 | ± | 0,94 |
|  | **Treatment 2 (W4)** | 9,25 | ± | 1,71 | 4,14 | ± | 0,12 | 7,90 | ± | 0,97 | 23,63 | ± | 1,34 |
|  | **Treatment 2 (W5)** | 8,70 | ± | 2,25 | 3,83 | ± | 0,63 | 5,80 | ± | 0,98 | 20,69 | ± | 3,75 |
|  | **Washout 1 (W6)** | 11,30 | ± | 0,44 | 5,22 | ± | 0,18 | 8,09 | ± | 0,88 | 27,16 | ± | 0,70 |
|  | **Washout 2 (W7)** | 11,22 | ± | 0,22 | 4,96 | ± | 0,02 | 7,69 | ± | 0,30 | 26,44 | ± | 0,52 |
| TC | **Control 1 (W1)** | 20,08 | ± | 2,36 | 7,55 | ± | 0,36 | 6,48 | ± | 0,30 | 36,18 | ± | 2,30 |
|  | **Control 2 (W2)** | 17,84 | ± | 1,74 | 7,93 | ± | 1,20 | 7,37 | ± | 1,63 | 35,60 | ± | 4,83 |
|  | **Treatment 1 (W3)** | 21,53 | ± | 0,62 | 11,17 | ± | 1,11 | 10,80 | ± | 0,54 | 46,22 | ± | 2,01 |
|  | **Treatment 2 (W4)** | 22,36 | ± | 0,46 | 12,40 | ± | 0,46 | 10,13 | ± | 0,61 | 47,57 | ± | 0,58 |
|  | **Treatment 2 (W5)** | 23,52 | ± | 1,47 | 12,95 | ± | 0,47 | 7,70 | ± | 0,48 | 46,82 | ± | 2,22 |
|  | **Washout 1 (W6)** | 23,50 | ± | 0,47 | 12,34 | ± | 1,05 | 9,80 | ± | 0,51 | 48,59 | ± | 1,09 |
|  | **Washout 2 (W7)** | 24,16 | ± | 1,14 | 11,15 | ± | 0,67 | 10,11 | ± | 0,41 | 48,45 | ± | 2,12 |
| DC | **Control 1 (W1)** | 24,67 | ± | 2,69 | 10,37 | ± | 0,66 | 7,37 | ± | 0,35 | 44,73 | ± | 3,10 |
|  | **Control 2 (W2)** | 21,26 | ± | 1,30 | 9,91 | ± | 0,67 | 7,57 | ± | 1,32 | 41,26 | ± | 3,47 |
|  | **Treatment 1 (W3)** | 24,04 | ± | 0,59 | 12,19 | ± | 0,99 | 11,01 | ± | 0,86 | 50,09 | ± | 2,28 |
|  | **Treatment 2 (W4)** | 24,77 | ± | 0,70 | 13,49 | ± | 0,60 | 10,47 | ± | 0,35 | 51,48 | ± | 1,50 |
|  | **Treatment 2 (W5)** | 27,40 | ± | 1,09 | 14,05 | ± | 0,68 | 8,15 | ± | 0,70 | 52,38 | ± | 2,40 |
|  | **Washout 1 (W6)** | 26,45 | ± | 0,94 | 13,45 | ± | 1,23 | 10,00 | ± | 0,55 | 53,00 | ± | 1,60 |
|  | **Washout 2 (W7)** | 24,84 | ± | 0,64 | 11,56 | ± | 0,51 | 10,21 | ± | 0,40 | 49,68 | ± | 1,47 |

Table S17. Short chain fatty acid (SCFA) production during the baby M-SHIME experiment (donor B, 1.5, 3 and 4.5 g/d SCF). Data represent geometric mean ± standard deviation (n=3 time points per week) of SCFA at different time points of the assay. PC = proximal colon; DC = distal colon; SCF = soluble corn fiber

|  |  | Short chain fatty acids (mmol/L) | | | | | | | | | | | | |
| --- | --- | --- | --- | --- | --- | --- | --- | --- | --- | --- | --- | --- | --- | --- |
| SCF |  | Week (W) | Acetate | | | Propionate | | | Butyrate | | | Total SCFA | | |
| 1.5 g/d | PC | Control 1 (W1) | 4.52 | ± | 0.45 | 0.47 | ± | 0.24 | 5.62 | ± | 1.75 | 14.93 | ± | 2.55 |
|  |  | Control 2 (W2) | 3.53 | ± | 0.22 | 0.24 | ± | 0.01 | 6.10 | ± | 0.11 | 15.42 | ± | 0.34 |
|  |  | Treatment 1 (W3) | 3.97 | ± | 0.44 | 0.30 | ± | 0.05 | 6.37 | ± | 0.26 | 17.32 | ± | 1.22 |
|  |  | Treatment 2 (W4) | 3.71 | ± | 0.59 | 0.15 | ± | 0.13 | 6.86 | ± | 0.95 | 18.28 | ± | 0.62 |
|  |  | Treatment 2 (W5) | 4.82 | ± | 0.57 | 0.30 | ± | 0.03 | 7.23 | ± | 0.73 | 18.47 | ± | 1.61 |
|  |  | Washout 1 (W6) | 4.67 | ± | 1.12 | 0.27 | ± | 0.06 | 7.74 | ± | 1.29 | 19.02 | ± | 2.47 |
|  |  | Washout 2 (W7) | 3.69 | ± | 0.72 | 0.18 | ± | 0.16 | 6.74 | ± | 0.46 | 17.31 | ± | 1.33 |
|  | DC | Control 1 (W1) | 19.47 | ± | 1.17 | 2.77 | ± | 0.02 | 8.45 | ± | 0.84 | 36.01 | ± | 1.44 |
|  |  | Control 2 (W2) | 15.55 | ± | 0.99 | 2.64 | ± | 0.18 | 10.50 | ± | 0.50 | 34.86 | ± | 1.51 |
|  |  | Treatment 1 (W3) | 17.91 | ± | 3.28 | 3.71 | ± | 0.94 | 10.78 | ± | 1.03 | 39.30 | ± | 5.59 |
|  |  | Treatment 2 (W4) | 20.80 | ± | 0.40 | 4.63 | ± | 0.04 | 12.32 | ± | 0.74 | 45.09 | ± | 0.32 |
|  |  | Treatment 2 (W5) | 16.74 | ± | 3.33 | 4.01 | ± | 0.77 | 10.47 | ± | 3.15 | 36.90 | ± | 9.15 |
|  |  | Washout 1 (W6) | 19.94 | ± | 1.18 | 4.28 | ± | 0.65 | 13.03 | ± | 0.54 | 44.25 | ± | 1.35 |
|  |  | Washout 2 (W7) | 17.42 | ± | 2.40 | 3.42 | ± | 0.42 | 12.31 | ± | 1.02 | 40.78 | ± | 4.32 |
| 3 g/d | PC | Control 1 (W1) | 8.78 | ± | 0.15 | 0.86 | ± | 0.06 | 6.90 | ± | 0.22 | 20.71 | ± | 0.59 |
|  |  | Control 2 (W2) | 12.42 | ± | 0.53 | 1.11 | ± | 0.18 | 9.19 | ± | 0.37 | 27.70 | ± | 0.94 |
|  |  | Treatment 1 (W3) | 13.32 | ± | 0.80 | 1.41 | ± | 0.05 | 9.74 | ± | 0.52 | 29.87 | ± | 1.17 |
|  |  | Treatment 2 (W4) | 14.89 | ± | 1.71 | 1.54 | ± | 0.23 | 12.25 | ± | 1.03 | 35.08 | ± | 3.41 |
|  |  | Treatment 2 (W5) | 13.31 | ± | 0.98 | 1.36 | ± | 0.12 | 11.85 | ± | 1.40 | 32.44 | ± | 2.73 |
|  |  | Washout 1 (W6) | 13.50 | ± | 0.93 | 1.62 | ± | 0.22 | 11.68 | ± | 1.56 | 33.34 | ± | 3.55 |
|  |  | Washout 2 (W7) | 12.75 | ± | 0.23 | 1.59 | ± | 0.03 | 10.70 | ± | 0.12 | 31.18 | ± | 0.16 |
|  | DC | Control 1 (W1) | 23.21 | ± | 3.29 | 2.86 | ± | 0.27 | 9.51 | ± | 0.45 | 40.60 | ± | 3.80 |
|  |  | Control 2 (W2) | 21.24 | ± | 2.99 | 2.77 | ± | 0.31 | 10.47 | ± | 0.81 | 39.93 | ± | 4.40 |
|  |  | Treatment 1 (W3) | 24.07 | ± | 4.33 | 4.53 | ± | 1.70 | 11.77 | ± | 1.46 | 46.08 | ± | 7.53 |
|  |  | Treatment 2 (W4) | 30.16 | ± | 3.35 | 6.33 | ± | 0.85 | 14.67 | ± | 0.74 | 57.51 | ± | 5.13 |
|  |  | Treatment 2 (W5) | 27.19 | ± | 1.50 | 5.77 | ± | 0.23 | 14.61 | ± | 0.89 | 53.88 | ± | 2.69 |
|  |  | Washout 1 (W6) | 25.76 | ± | 7.56 | 4.98 | ± | 2.69 | 13.93 | ± | 2.68 | 51.62 | ± | 13.37 |
|  |  | Washout 2 (W7) | 19.40 | ± | 0.25 | 2.77 | ± | 0.03 | 11.71 | ± | 0.10 | 40.44 | ± | 0.15 |
| 4.5 g/d | PC | Control 1 (W1) | 7.86 | ± | 0.22 | 0.41 | ± | 0.03 | 7.43 | ± | 0.30 | 20.46 | ± | 0.62 |
|  |  | Control 2 (W2) | 8.49 | ± | 0.36 | 0.62 | ± | 0.15 | 7.64 | ± | 0.34 | 21.74 | ± | 0.16 |
|  |  | Treatment 1 (W3) | 9.32 | ± | 0.36 | 0.87 | ± | 0.09 | 8.40 | ± | 1.24 | 24.47 | ± | 2.47 |
|  |  | Treatment 2 (W4) | 12.08 | ± | 2.06 | 0.79 | ± | 0.07 | 11.47 | ± | 1.34 | 31.72 | ± | 4.40 |
|  |  | Treatment 2 (W5) | 11.50 | ± | 0.77 | 0.65 | ± | 0.02 | 10.65 | ± | 0.39 | 29.01 | ± | 0.92 |
|  |  | Washout 1 (W6) | 10.81 | ± | 2.15 | 0.80 | ± | 0.16 | 9.53 | ± | 0.73 | 27.05 | ± | 2.81 |
|  |  | Washout 2 (W7) | 10.33 | ± | 0.66 | 1.00 | ± | 0.00 | 9.15 | ± | 0.43 | 26.22 | ± | 1.14 |
|  | DC | Control 1 (W1) | 18.84 | ± | 0.77 | 2.69 | ± | 0.11 | 10.67 | ± | 0.70 | 37.72 | ± | 0.21 |
|  |  | Control 2 (W2) | 19.30 | ± | 0.72 | 3.27 | ± | 0.16 | 10.74 | ± | 0.11 | 39.05 | ± | 0.90 |
|  |  | Treatment 1 (W3) | 24.54 | ± | 4.83 | 5.32 | ± | 1.74 | 13.42 | ± | 2.25 | 49.57 | ± | 9.57 |
|  |  | Treatment 2 (W4) | 28.75 | ± | 0.47 | 5.93 | ± | 0.41 | 15.56 | ± | 0.51 | 57.72 | ± | 1.00 |
|  |  | Treatment 2 (W5) | 33.70 | ± | 2.92 | 6.51 | ± | 0.99 | 17.19 | ± | 0.45 | 64.77 | ± | 4.16 |
|  |  | Washout 1 (W6) | 31.17 | ± | 10.42 | 6.70 | ± | 2.85 | 16.10 | ± | 3.41 | 61.38 | ± | 17.14 |
|  |  | Washout 2 (W7) | 21.50 | ± | 0.86 | 4.04 | ± | 0.24 | 12.87 | ± | 0.09 | 45.26 | ± | 1.00 |

Table S18. Short chain fatty acid (SCFA) production during the baby M-SHIME experiment (donor C and D, 3 g/d SCF). Data represent geometric mean ± standard deviation (n=3 time points per week) of SCFA at different time points of the assay. PC = proximal colon; DC = distal colon; SCF = soluble corn fiber

|  |  |  | SCFA (mmol/L) | | | | | | | | | | | | |
| --- | --- | --- | --- | --- | --- | --- | --- | --- | --- | --- | --- | --- | --- | --- | --- |
| Donor |  | Week | Acetate | | | Propionate | | | Butyrate | | | Total SCFA | | | |
| Baby C | PC | Control 1 (W1) | 20.74 | ± | 1.10 | 11.01 | ± | 0.76 | 8.09 | ± | 0.45 | 42.08 | ± | 2.28 |  |
|  |  | Control 2 (W2) | 21.89 | ± | 1.40 | 10.80 | ± | 1.19 | 8.41 | ± | 1.45 | 43.42 | ± | 4.14 |  |
|  |  | Treatment 1 (W3) | 34.47 | ± | 0.40 | 22.07 | ± | 1.66 | 13.25 | ± | 1.09 | 72.05 | ± | 2.80 |  |
|  |  | Treatment 2 (W4) | 33.84 | ± | 2.34 | 20.05 | ± | 1.45 | 13.75 | ± | 1.64 | 69.83 | ± | 5.70 |  |
|  |  | Treatment 2 (W5) | 32.47 | ± | 1.76 | 21.11 | ± | 1.15 | 14.69 | ± | 0.31 | 70.61 | ± | 2.21 |  |
|  | DC | Control 1 (W1) | 24.51 | ± | 3.04 | 13.09 | ± | 1.65 | 10.52 | ± | 0.94 | 51.10 | ± | 6.06 |  |
|  |  | Control 2 (W2) | 22.71 | ± | 1.10 | 11.16 | ± | 0.79 | 9.33 | ± | 0.91 | 45.80 | ± | 2.75 |  |
|  |  | Treatment 1 (W3) | 36.76 | ± | 2.69 | 26.47 | ± | 1.93 | 11.60 | ± | 0.39 | 77.21 | ± | 4.96 |  |
|  |  | Treatment 2 (W4) | 40.27 | ± | 0.77 | 28.91 | ± | 0.80 | 13.80 | ± | 0.96 | 85.50 | ± | 2.51 |  |
|  |  | Treatment 2 (W5) | 37.59 | ± | 1.63 | 27.18 | ± | 0.70 | 13.38 | ± | 0.62 | 80.60 | ± | 2.90 |  |
| Baby D | PC | Control 1 (W1) | 23.25 | ± | 2.79 | 9.13 | ± | 0.85 | 11.00 | ± | 0.53 | 46.30 | ± | 4.07 |  |
|  |  | Control 2 (W2) | 22.95 | ± | 2.82 | 9.51 | ± | 1.02 | 11.72 | ± | 0.28 | 47.25 | ± | 4.18 |  |
|  |  | Treatment 1 (W3) | 28.55 | ± | 0.86 | 18.46 | ± | 5.43 | 15.77 | ± | 2.55 | 65.54 | ± | 4.84 |  |
|  |  | Treatment 2 (W4) | 32.97 | ± | 3.68 | 28.09 | ± | 2.68 | 14.41 | ± | 1.81 | 78.40 | ± | 7.96 |  |
|  |  | Treatment 2 (W5) | 37.64 | ± | 1.13 | 28.96 | ± | 1.65 | 14.91 | ± | 0.50 | 84.70 | ± | 3.19 |  |
|  | DC | Control 1 (W1) | 24.43 | ± | 0.82 | 9.18 | ± | 0.30 | 11.51 | ± | 0.42 | 48.40 | ± | 1.60 |  |
|  |  | Control 2 (W2) | 26.41 | ± | 2.87 | 9.49 | ± | 1.12 | 11.34 | ± | 1.10 | 52.94 | ± | 5.69 |  |
|  |  | Treatment 1 (W3) | 33.86 | ± | 3.11 | 20.80 | ± | 3.98 | 16.29 | ± | 0.70 | 75.14 | ± | 7.83 |  |
|  |  | Treatment 2 (W4) | 37.69 | ± | 4.26 | 29.81 | ± | 3.97 | 13.10 | ± | 2.36 | 85.79 | ± | 11.1 |  |
|  |  | Treatment 2 (W5) | 40.95 | ± | 1.93 | 31.49 | ± | 0.96 | 14.29 | ± | 0.43 | 92.41 | ± | 2.26 |  |

Table S19. Short chain fatty acid (SCFA) production during the M-SHIME experiment (adult A, 3 g/d SCF; adult B 8.5 g/d). Data represent geometric mean ± standard deviation (n=3 time points per week) of SCFA at different time points of the assay. PC = proximal colon; DC = distal colon; AC = ascending colon; TC= transverse colon; SCF = soluble corn fiber

|  |  |  |  | Short chain fatty acids (mmol/L) | | | | | | | | | | | |
| --- | --- | --- | --- | --- | --- | --- | --- | --- | --- | --- | --- | --- | --- | --- | --- |
| SCF | Donor |  | Week | Acetate | | | Propionate | | | Butyrate | | | Total SCFA | | |
| 3 g/d | Adult A | PC | Control 1 (W1) | 26.89 | ± | 3.18 | 7.22 | ± | 0.82 | 8.47 | ± | 0.32 | 44.55 | ± | 4.32 |
|  |  |  | Control 2 (W2) | 25.60 | ± | 0.31 | 7.46 | ± | 0.21 | 9.14 | ± | 0.38 | 44.19 | ± | 0.80 |
|  |  |  | Treatment 1 (W3) | 28.23 | ± | 0.81 | 9.20 | ± | 0.56 | 10.64 | ± | 2.86 | 50.17 | ± | 3.47 |
|  |  |  | Treatment 2 (W4) | 25.68 | ± | 1.52 | 9.58 | ± | 1.04 | 13.67 | ± | 0.81 | 50.97 | ± | 2.46 |
|  |  |  | Treatment 2 (W5) | 23.88 | ± | 1.18 | 12.68 | ± | 0.94 | 12.91 | ± | 2.27 | 51.80 | ± | 2.54 |
|  |  |  | Washout 1 (W6) | 22.32 | ± | 0.65 | 7.43 | ± | 0.75 | 15.15 | ± | 2.40 | 47.30 | ± | 4.04 |
|  |  |  | Washout 2 (W7) | 22.76 | ± | 0.62 | 6.31 | ± | 0.60 | 12.42 | ± | 0.92 | 43.55 | ± | 0.95 |
|  |  | DC | Control 1 (W1) | 44.14 | ± | 1.00 | 9.94 | ± | 0.08 | 10.45 | ± | 0.21 | 68.08 | ± | 1.12 |
|  |  |  | Control 2 (W2) | 42.58 | ± | 2.72 | 10.19 | ± | 0.62 | 10.17 | ± | 0.88 | 66.47 | ± | 4.42 |
|  |  |  | Treatment 1 (W3) | 54.19 | ± | 2.11 | 16.74 | ± | 1.10 | 11.41 | ± | 1.23 | 85.96 | ± | 3.57 |
|  |  |  | Treatment 2 (W4) | 53.91 | ± | 0.26 | 17.63 | ± | 0.95 | 14.38 | ± | 0.45 | 89.86 | ± | 1.31 |
|  |  |  | Treatment 2 (W5) | 50.79 | ± | 1.21 | 19.39 | ± | 0.28 | 12.42 | ± | 1.41 | 86.55 | ± | 2.79 |
|  |  |  | Washout 1 (W6) | 44.69 | ± | 5.42 | 12.92 | ± | 2.99 | 14.81 | ± | 1.35 | 76.88 | ± | 10.18 |
|  |  |  | Washout 2 (W7) | 44.21 | ± | 2.06 | 9.58 | ± | 0.51 | 11.30 | ± | 1.14 | 68.70 | ± | 1.09 |
| 8.5 g/d | Adult B | AC | Control 1 (W1) | 15,59 | ± | 1,58 | 8,67 | ± | 0,30 | 8,48 | ± | 1,42 | 34,48 | ± | 3,41 |
|  |  |  | Control 2 (W2) | 14,87 | ± | 2,17 | 8,00 | ± | 0,60 | 8,71 | ± | 0,32 | 33,26 | ± | 3,04 |
|  |  |  | Treatment 1 (W3) | 27,26 | ± | 12,06 | 9,02 | ± | 1,33 | 12,87 | ± | 4,04 | 50,65 | ± | 17,07 |
|  |  |  | Treatment 2 (W4) | 28,23 | ± | 2,32 | 14,54 | ± | 1,08 | 19,24 | ± | 1,14 | 63,45 | ± | 0,53 |
|  |  |  | Treatment 2 (W5) | 24,03 | ± | 3,57 | 15,12 | ± | 1,79 | 18,06 | ± | 0,99 | 58,59 | ± | 6,17 |
|  |  |  | Washout 1 (W6) | 17,44 | ± | 8,45 | 11,72 | ± | 5,50 | 14,18 | ± | 3,73 | 45,00 | ± | 17,34 |
|  |  |  | Washout 2 (W7) | 12,64 | ± | 2,03 | 7,04 | ± | 0,83 | 9,74 | ± | 0,89 | 31,14 | ± | 3,86 |
|  |  | TC | Control 1 (W1) | 23,33 | ± | 1,91 | 10,42 | ± | 0,99 | 11,20 | ± | 0,87 | 47,03 | ± | 3,63 |
|  |  |  | Control 2 (W2) | 22,04 | ± | 0,74 | 10,28 | ± | 0,20 | 11,57 | ± | 0,30 | 46,01 | ± | 0,86 |
|  |  |  | Treatment 1 (W3) | 35,48 | ± | 15,60 | 16,54 | ± | 6,58 | 15,23 | ± | 4,45 | 69,08 | ± | 26,43 |
|  |  |  | Treatment 2 (W4) | 51,87 | ± | 2,07 | 26,65 | ± | 1,98 | 22,75 | ± | 0,77 | 102,90 | ± | 2,82 |
|  |  |  | Treatment 2 (W5) | 51,45 | ± | 0,91 | 32,24 | ± | 0,86 | 22,89 | ± | 0,35 | 108,22 | ± | 1,39 |
|  |  |  | Washout 1 (W6) | 36,63 | ± | 11,78 | 26,83 | ± | 8,86 | 19,81 | ± | 2,48 | 85,15 | ± | 22,83 |
|  |  |  | Washout 2 (W7) | 19,14 | ± | 1,29 | 10,81 | ± | 0,91 | 12,84 | ± | 1,19 | 44,79 | ± | 2,91 |
|  |  | DC | Control 1 (W1) | 30,12 | ± | 1,00 | 10,46 | ± | 0,90 | 10,00 | ± | 0,29 | 56,13 | ± | 1,75 |
|  |  |  | Control 2 (W2) | 28,51 | ± | 0,90 | 10,63 | ± | 0,30 | 10,63 | ± | 0,09 | 54,73 | ± | 1,36 |
|  |  |  | Treatment 1 (W3) | 42,23 | ± | 14,52 | 17,45 | ± | 6,51 | 12,34 | ± | 2,05 | 77,68 | ± | 24,07 |
|  |  |  | Treatment 2 (W4) | 68,36 | ± | 2,57 | 30,84 | ± | 1,84 | 18,09 | ± | 0,84 | 126,20 | ± | 4,01 |
|  |  |  | Treatment 2 (W5) | 62,65 | ± | 0,73 | 33,62 | ± | 0,09 | 18,75 | ± | 0,27 | 124,19 | ± | 0,43 |
|  |  |  | Washout 1 (W6) | 51,74 | ± | 8,44 | 29,53 | ± | 5,34 | 17,67 | ± | 0,33 | 107,63 | ± | 14,73 |
|  |  |  | Washout 2 (W7) | 27,16 | ± | 0,97 | 12,46 | ± | 1,41 | 12,87 | ± | 0,85 | 57,24 | ± | 3,81 |

Table S20. Short chain fatty acid (SCFA) production during the M-SHIME experiment (elderly, 8.5 g/d). Data represent geometric mean ± standard deviation (n=3 time points per week) of SCFA at different time points of the assay. AC = ascending colon; TC = transverse colon; DC = distal colon.

|  |  | SCFA (mmol/L) | | | | | | | | | | | |
| --- | --- | --- | --- | --- | --- | --- | --- | --- | --- | --- | --- | --- | --- |
|  |  | Acetate | | | Propionate | | | Butyrate | | | Total SCFA | | |
| AC | Control 1 (W1) | 18.70 | ± | 1.32 | 8.74 | ± | 0.78 | 18.17 | ± | 0.13 | 48.19 | ± | 2.23 |
|  | Control 2 (W2) | 18.00 | ± | 1.69 | 9.16 | ± | 0.75 | 21.39 | ± | 1.27 | 51.35 | ± | 3.56 |
|  | Treatment 1 (W3) | 20.78 | ± | 1.85 | 9.87 | ± | 0.67 | 26.74 | ± | 4.51 | 60.12 | ± | 4.53 |
|  | Treatment 2 (W4) | 28.49 | ± | 12.00 | 15.13 | ± | 9.09 | 34.57 | ± | 3.02 | 80.87 | ± | 22.57 |
|  | Treatment 2 (W5) | 19.46 | ± | 1.18 | 9.69 | ± | 0.95 | 22.03 | ± | 2.74 | 53.91 | ± | 3.65 |
|  | Washout 1 (W6) | 21.83 | ± | 2.79 | 9.70 | ± | 1.18 | 38.32 | ± | 1.30 | 72.35 | ± | 2.95 |
|  | Washout 2 (W7) | 20.57 | ± | 3.26 | 8.68 | ± | 0.33 | 17.33 | ± | 0.88 | 48.97 | ± | 2.20 |
| TC | Control 1 (W1) | 20.71 | ± | 0.57 | 10.54 | ± | 1.07 | 17.44 | ± | 0.32 | 51.56 | ± | 1.99 |
|  | Control 2 (W2) | 21.45 | ± | 0.66 | 11.94 | ± | 0.39 | 19.26 | ± | 1.66 | 55.73 | ± | 2.10 |
|  | Treatment 1 (W3) | 34.06 | ± | 7.47 | 21.34 | ± | 6.68 | 25.04 | ± | 7.30 | 83.42 | ± | 21.51 |
|  | Treatment 2 (W4) | 39.06 | ± | 14.51 | 22.39 | ± | 10.93 | 36.74 | ± | 2.12 | 101.08 | ± | 27.71 |
|  | Treatment 2 (W5) | 23.30 | ± | 0.27 | 12.90 | ± | 0.97 | 19.61 | ± | 3.36 | 58.89 | ± | 2.42 |
|  | Washout 1 (W6) | 49.85 | ± | 11.10 | 27.03 | ± | 1.89 | 41.25 | ± | 6.07 | 121.08 | ± | 18.54 |
|  | Washout 2 (W7) | 22.66 | ± | 1.03 | 10.81 | ± | 0.63 | 15.06 | ± | 1.21 | 51.35 | ± | 2.86 |
| DC | Control 1 (W1) | 23.51 | ± | 1.98 | 11.39 | ± | 1.31 | 15.84 | ± | 0.29 | 56.16 | ± | 4.20 |
|  | Control 2 (W2) | 22.55 | ± | 3.69 | 13.47 | ± | 1.01 | 17.14 | ± | 1.75 | 58.43 | ± | 6.40 |
|  | Treatment 1 (W3) | 39.21 | ± | 15.57 | 24.77 | ± | 11.18 | 21.39 | ± | 5.72 | 89.44 | ± | 32.10 |
|  | Treatment 2 (W4) | 61.96 | ± | 1.26 | 45.47 | ± | 1.50 | 33.48 | ± | 1.85 | 144.88 | ± | 2.62 |
|  | Treatment 2 (W5) | 24.24 | ± | 1.80 | 14.10 | ± | 2.88 | 17.55 | ± | 2.83 | 60.79 | ± | 7.08 |
|  | Washout 1 (W6) | 59.70 | ± | 23.94 | 45.82 | ± | 11.24 | 36.51 | ± | 5.83 | 146.07 | ± | 44.62 |
|  | Washout 2 (W7) | 23.83 | ± | 4.16 | 12.33 | ± | 0.74 | 14.51 | ± | 0.45 | 54.95 | ± | 4.97 |

**
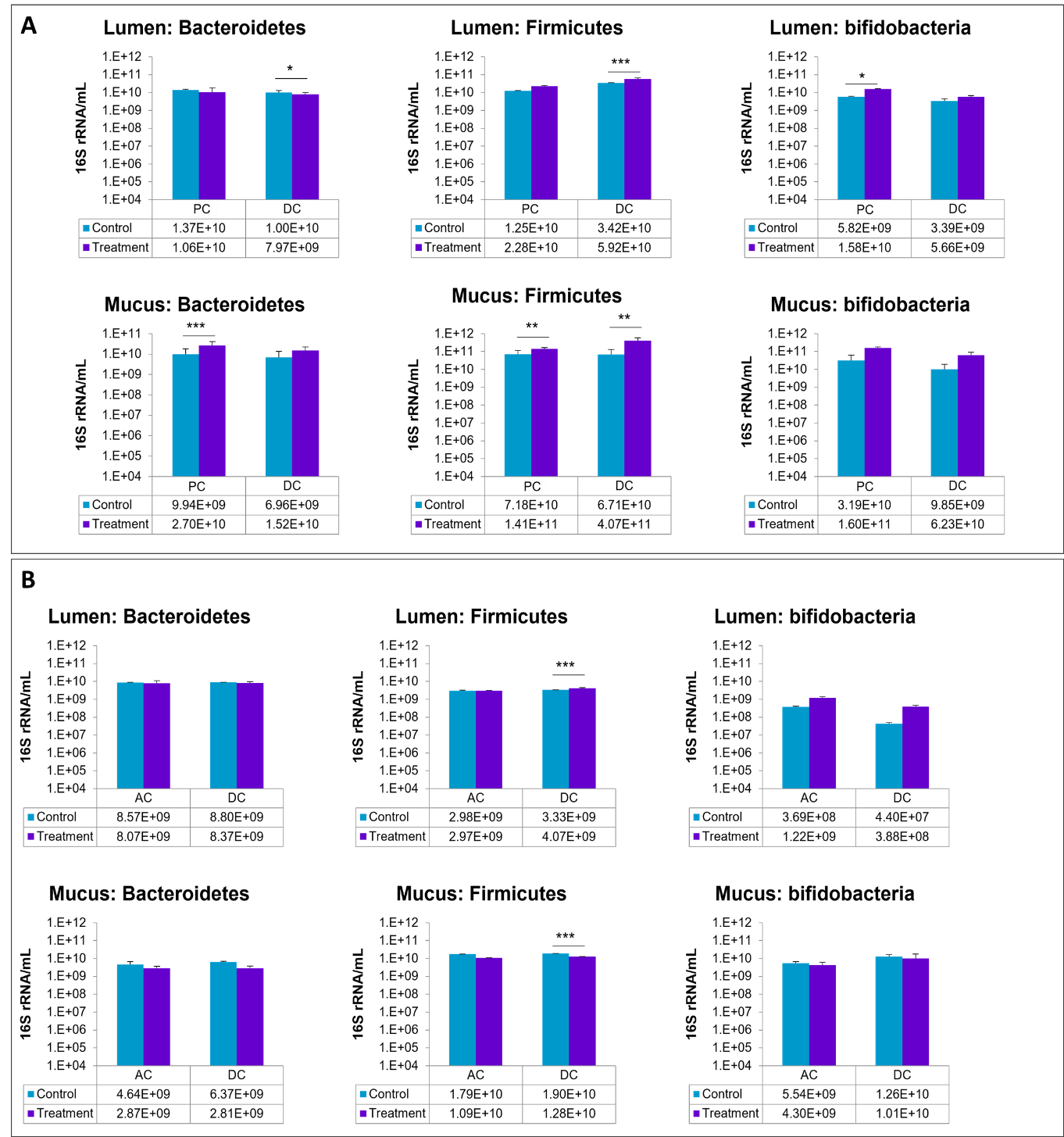
**

Fig. S3 Luminal and mucosal microbial community composition during the control and treatment periods in the adult M-SHIME^®^ experiment as assessed by qPCR for adult A (3 g/d SCF, panel A) and adult B (8.5 g/d SCF, panel B). Bars represent geometric mean ± standard deviation of three sampling points per week (n=6 for control and n=9 for treatment) and three technical replicates of the qPCR technique. Data are shown as mean ± standard deviation. Data were analyzed using Tukey's multiple comparison's test. * p <0.05, **p <0.01, *** p <0.001. AC = ascending colon; DC = distal colon; M-SHIME^®^ = mucosal simulator of the human intestinal microbial ecosystem; PC = proximal colon; SCF = soluble corn fiber

**
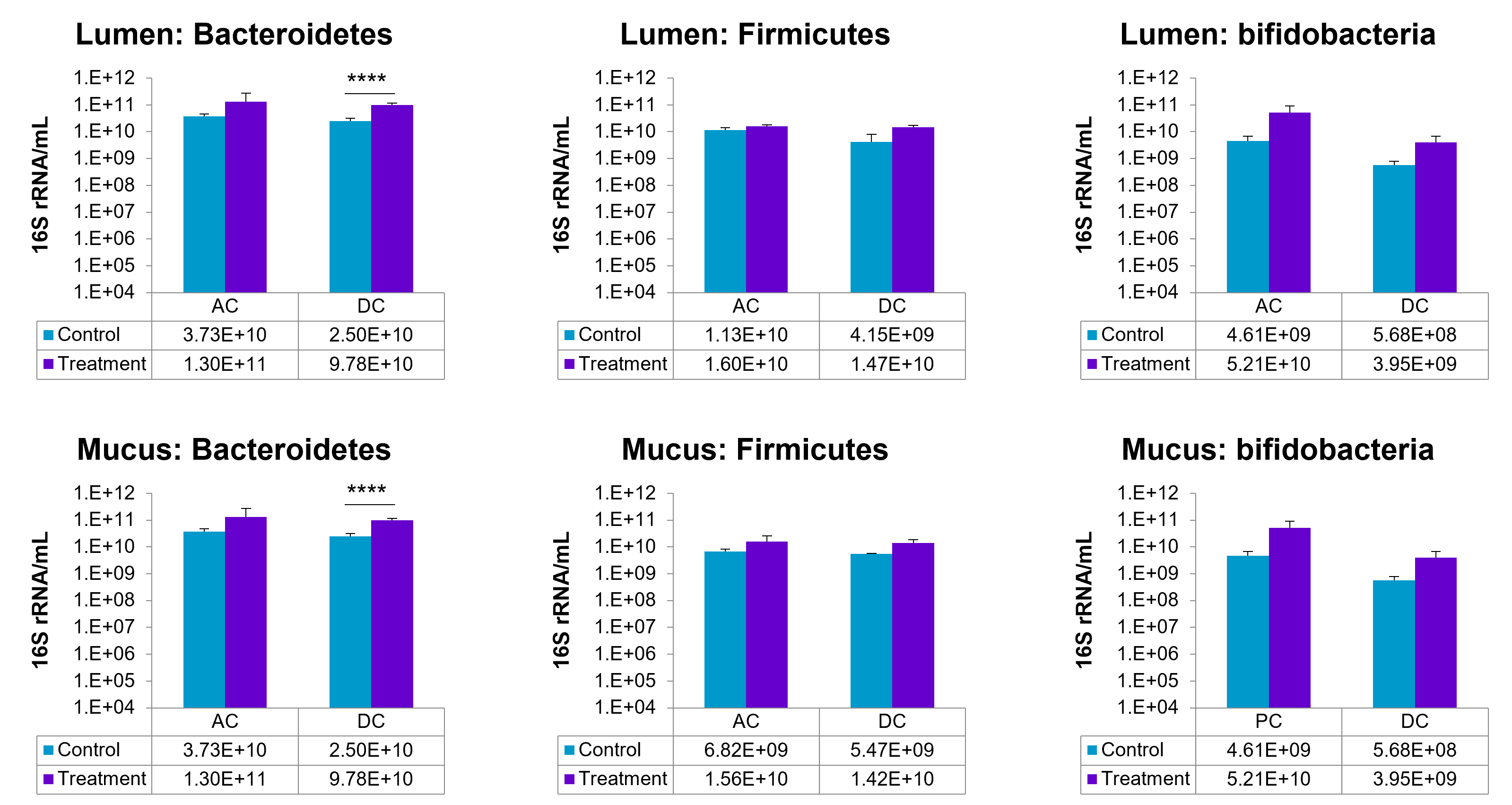
**Fig. S4 Luminal and mucosal microbial community composition during the control and treatment periods (8.5 g/d SCF) in the elderly M-SHIME^®^ experiment as assessed by qPCR. Bars represent geometric mean ± standard deviation of three sampling points per week (n=6 for control and n=9 for treatment) and three technical replicates of the qPCR technique. Data were analyzed using Tukey's multiple comparison's test. **** p <0.0001. AC = ascending colon; DC = distal colon; M-SHIME^®^ = mucosal simulator of the human intestinal microbial ecosystem; SCF = soluble corn fiber

**
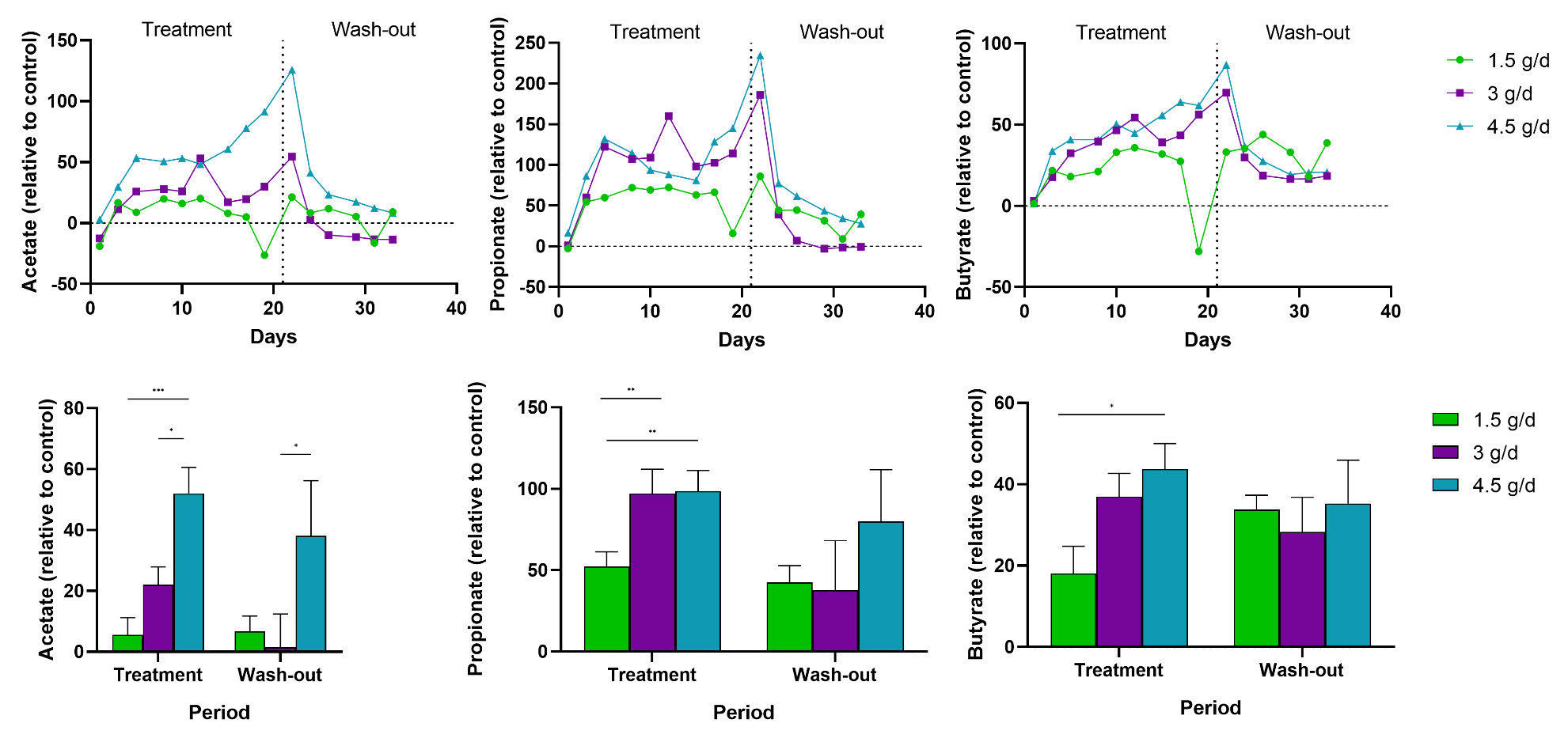
**

Fig. S5 Changes in SCFA levels in the DC during the treatment (Baby B, 1.5 g/d, 3 g/d, and 4.5 g/d SCF) and washout periods in the baby M-SHIME^®^. The top three graphs show changes in SCFA levels over time relative to the control period. The bottom three graphs show the mean SCFA level for the treatment (3 sampling times per week, 3 weeks, n=9) or washout (2 sampling points per week, 2 weeks, n=6) period relative to the control period; error bars represent standard error of the mean. Differences in SCFA levels between SCF doses were analyzed using a two-way ANOVA with Dunnett's multiple comparison test. * p <0.05, **p ≤0.01, *** p <0.001. Samples were assayed in triplicate. ANOVA = analysis of variance; DC = distal colon; M-SHIME^®^ = mucosal simulator of the human intestinal microbial ecosystem; SCF = soluble corn fiber; SCFA, short-chain fatty acid

**A**

**
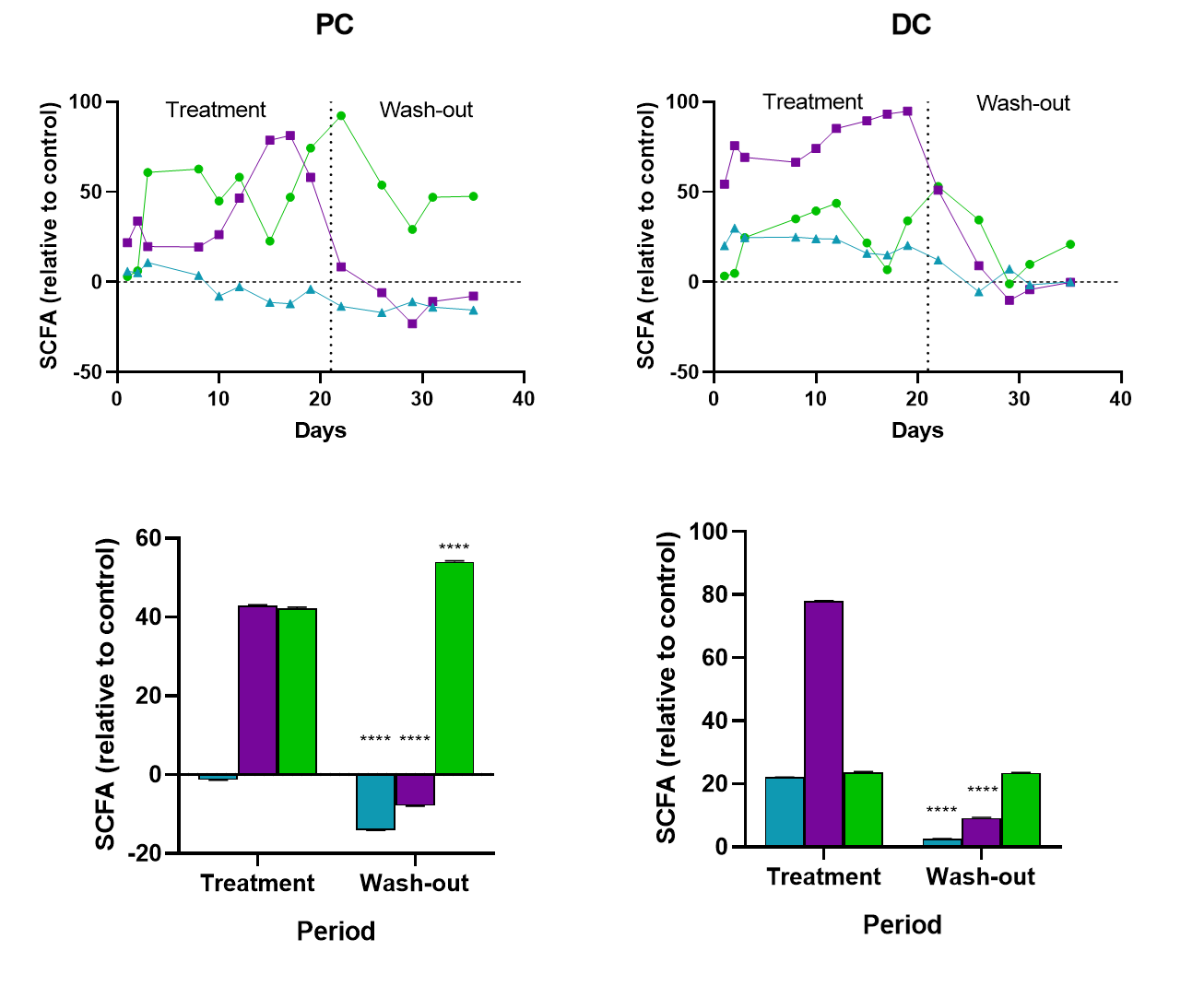
**

**B**

**
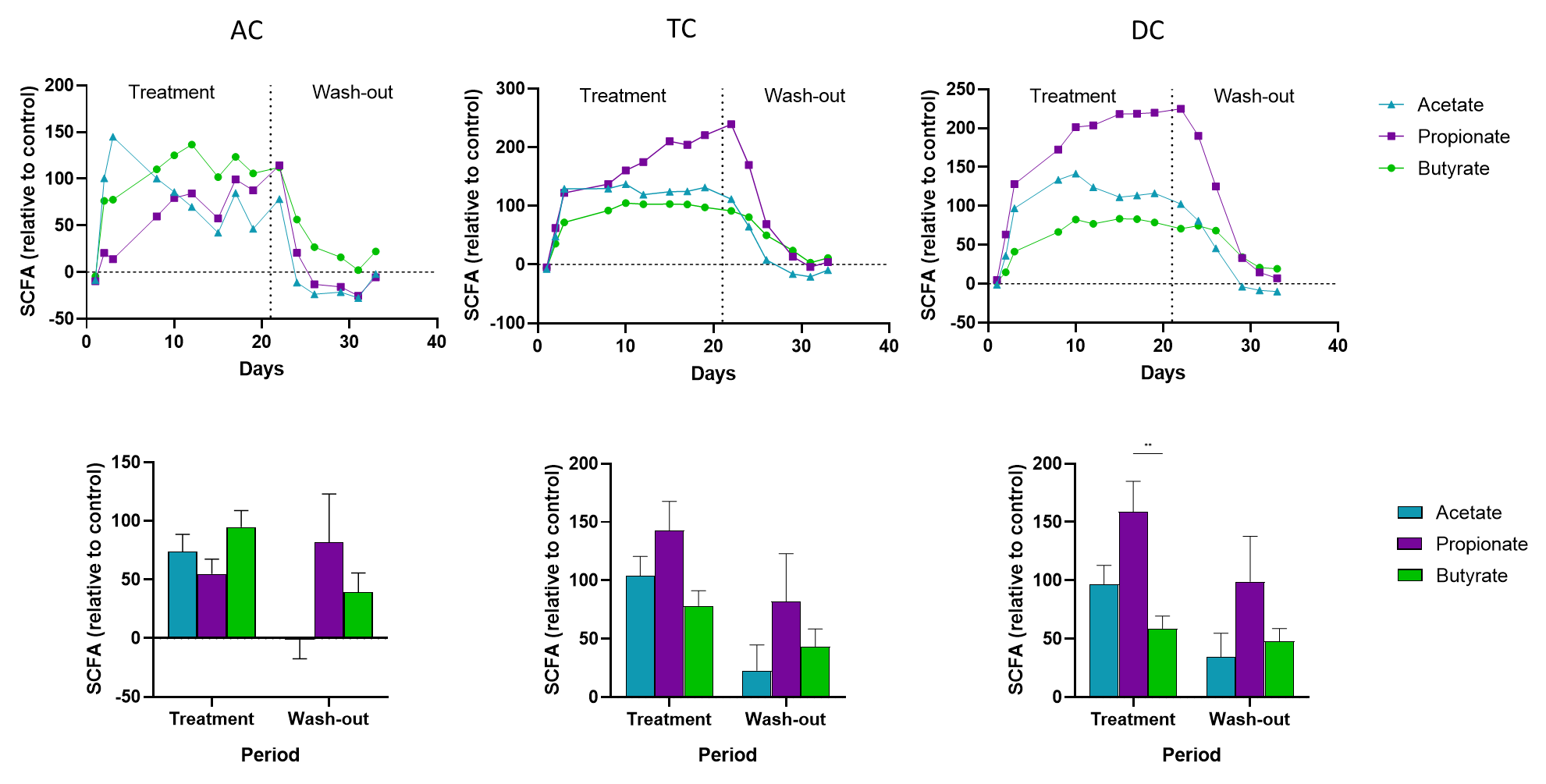
**

Fig. S6 Changes in SCFA levels in the PC and DC for adult A (3 g/d SCF) (A) and in the AC, TC and DC for adult B (8.5 g/d SCF)(B) during the treatment and washout periods in the adult M-SHIME^®^. The top graphs show changes in SCFA levels over time relative to the control period. The bottom graphs show the SCFA level for the treatment or washout period relative to the control period. Bars represent the average ± standard error of the mean (n = 9 sampling points for the treatment period and n = 6 sampling points for the washout period. Differences between SCFA levels were analyzed using a two-way ANOVA with Dunnett's multiple comparison test. **p <0.01. ANOVA = analysis of variance; AC = ascending colon; DC = distal colon; M-SHIME^®^ = mucosal simulator of the human intestinal microbial ecosystem; SCF = soluble corn fiber; SCFA, short-chain fatty acid

**
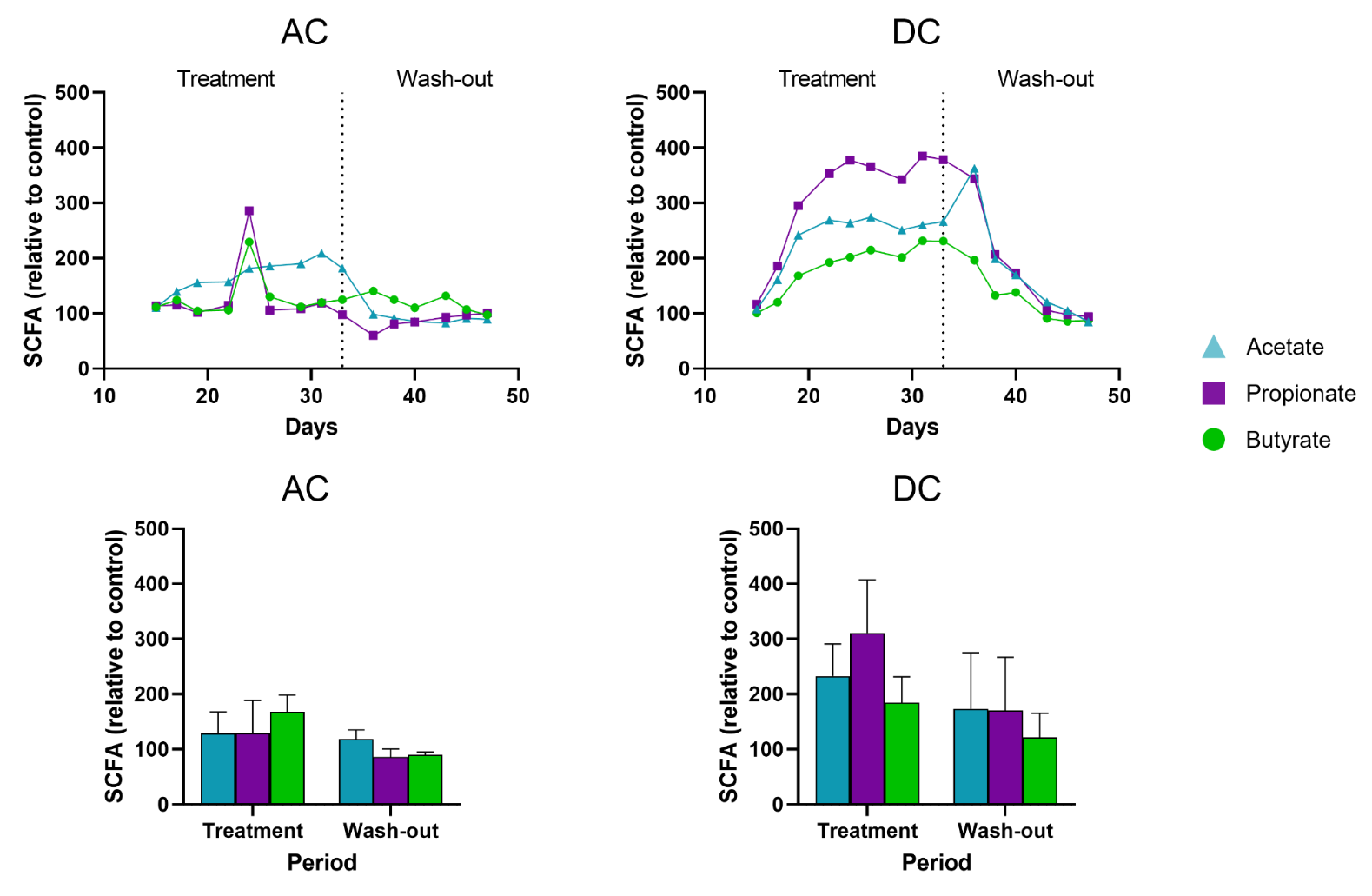
**

Fig. S7. Changes in SCFA levels in the AC and DC during the treatment (8.5 g/d SCF) and washout periods in the elderly M-SHIME^®^. The top two graphs show changes in SCFA levels over time relative to the control period. The bottom graphs show the SCFA level for the treatment or washout period relative to the control period. Bars represent the average ± standard error of the mean (n = 9 sampling points for the treatment period and n = 6 sampling points for the washout period. Differences between SCFA levels were analyzed using a two-way ANOVA with Dunnett's multiple comparison test. Samples were assayed in triplicate. ANOVA = analysis of variance; AC = ascending colon; DC = distal colon; M-SHIME^®^ = mucosal simulator of the human intestinal microbial ecosystem; SCF = soluble corn fiber; SCFA, short-chain fatty acid

**References**

1. Molly, K.; Vande Woestyne, M.; Verstraete, W. Development of a 5-Step Multi-Chamber Reactor as a Simulation of the Human Intestinal Microbial Ecosystem. *Applied microbiology and biotechnology* **1993**, *39*, 254–258.

2. Van den Abbeele, P.; Roos, S.; Eeckhaut, V.; MacKenzie, D.A.; Derde, M.; Verstraete, W.; Marzorati, M.; Possemiers, S.; Vanhoecke, B.; Van Immerseel, F. Incorporating a Mucosal Environment in a Dynamic Gut Model Results in a More Representative Colonization by Lactobacilli. *Microbial biotechnology* **2012**, *5*, 106–115.

3. Daguet, D.; Pinheiro, I.; Verhelst, A.; Possemiers, S.; Marzorati, M. Arabinogalactan and Fructooligosaccharides Improve the Gut Barrier Function in Distinct Areas of the Colon in the Simulator of the Human Intestinal Microbial Ecosystem. *Journal of Functional Foods* **2016**, *20*, 369–379, doi:https://doi.org/10.1016/j.jff.2015.11.005.

4. Marzorati, M.; Abbeele, P.V.D.; Bubeck, S.S.; Bayne, T.; Krishnan, K.; Young, A.; Mehta, D.; DeSouza, A. Bacillus Subtilis HU58 and Bacillus Coagulans SC208 Probiotics Reduced the Effects of Antibiotic-Induced Gut Microbiome Dysbiosis in An M-SHIME((R)) Model. *Microorganisms* **2020**, *8*, doi:10.3390/microorganisms8071028.

5. Ghyselinck, J.; Verstrepen, L.; Moens, F.; Abbeele, P.V. den; Said, J.; Smith, B.; Bjarnason, I.; Basit, A.W.; Gaisford, S. A 4-Strain Probiotic Supplement Influences Gut Microbiota Composition and Gut Wall Function in Patients with Ulcerative Colitis. *International Journal of Pharmaceutics* **2020**, *587*, 119648, doi:https://doi.org/10.1016/j.ijpharm.2020.119648.

6. Boon, N.; Top, E.M.; Verstraete, W.; Siciliano, S.D. Bioaugmentation as a Tool to Protect the Structure and Function of an Activated-Sludge Microbial Community against a 3-Chloroaniline Shock Load. *Appl Environ Microbiol* **2003**, *69*, 1511–1520, doi:10.1128/aem.69.3.1511-1520.2003.

7. Nadkarni, M.A.; Martin, F.E.; Jacques, N.A.; Hunter, N. Determination of Bacterial Load by Real-Time PCR Using a Broad-Range (Universal) Probe and Primers Set. *Microbiology* 2002, *148*, 257–266.

8. Rinttilä, T.; Kassinen, A.; Malinen, E.; Krogius, L.; Palva, A. Development of an Extensive Set of 16S RDNA-Targeted Primers for Quantification of Pathogenic and Indigenous Bacteria in Faecal Samples by Real-Time PCR. *Journal of Applied Microbiology* **2004**, *97*, 1166–1177, doi:10.1111/j.1365-2672.2004.02409.x.

9. Guo, X.; Xia, X.; Tang, R.; Zhou, J.; Zhao, H.; Wang, K. Development of a Real-Time PCR Method for Firmicutes and Bacteroidetes in Faeces and Its Application to Quantify Intestinal Population of Obese and Lean Pigs. *Lett Appl Microbiol* **2008**, *47*, 367–373, doi:10.1111/j.1472-765X.2008.02408.x.

10. Furet, J.P.; Firmesse, O.; Gourmelon, M.; Bridonneau, C.; Tap, J.; Mondot, S.; Doré, J.; Corthier, G. Comparative Assessment of Human and Farm Animal Faecal Microbiota Using Real-Time Quantitative PCR. *FEMS Microbiol Ecol* **2009**, *68*, 351–362, doi:10.1111/j.1574-6941.2009.00671.x.

11. Metsalu, T.; Vilo, J. ClustVis: A Web Tool for Visualizing Clustering of Multivariate Data Using Principal Component Analysis and Heatmap. *Nucleic acids research* **2015**, *43*, W566–W570.
